# The genome sequence of *Barbarea vulgaris* facilitates the study of ecological biochemistry

**DOI:** 10.1038/srep40728

**Published:** 2017-01-17

**Authors:** Stephen L. Byrne, Pernille Østerbye Erthmann, Niels Agerbirk, Søren Bak, Thure Pavlo Hauser, Istvan Nagy, Cristiana Paina, Torben Asp

**Affiliations:** 1 Department of Molecular Biology and Genetics, Aarhus University, Forsøgsvej 1, 4200 Slagelse, Denmark; 2 Crop Science Department, Teagasc, Oak Park, Ireland; 3 Department of Plant and Environmental Sciences and Copenhagen Plant Science Center, University of Copenhagen, Thorvaldsensvej 40, 1871 Frederiksberg C, Denmark

## Abstract

The genus *Barbarea* has emerged as a model for evolution and ecology of plant defense compounds, due to its unusual glucosinolate profile and production of saponins, unique to the Brassicaceae. One species, *B. vulgaris*, includes two ‘types’, G-type and P-type that differ in trichome density, and their glucosinolate and saponin profiles. A key difference is the stereochemistry of hydroxylation of their common phenethylglucosinolate backbone, leading to epimeric glucobarbarins. Here we report a draft genome sequence of the G-type, and re-sequencing of the P-type for comparison. This enables us to identify candidate genes underlying glucosinolate diversity, trichome density, and study the genetics of biochemical variation for glucosinolate and saponins. *B. vulgaris* is resistant to the diamondback moth, and may be exploited for “dead-end” trap cropping where glucosinolates stimulate oviposition and saponins deter larvae to the extent that they die. The *B. vulgaris* genome will promote the study of mechanisms in ecological biochemistry to benefit crop resistance breeding.

The crucifer family (Brassicaceae) is a large plant family containing several important cultivated species, such as oilseed rape, mustards and the many cabbages, as well as the general model plant *Arabidopsis thaliana*. Characteristic for crucifers is their content of glucosinolates, a group of sulfur and nitrogen containing metabolites derived from amino acids. Glucosinolates constitute the major group of defense compounds in the family, with large structural diversity among species and higher taxa^1^. Glucosinolates are hydrolysed by myrosinases upon tissue damage, releasing diverse but generally toxic compounds depending on the specific glucosinolate structure^2^, and thereby act as phytoanticipins. Indole phytoalexins are also widespread in the family^3^. In addition to these general crucifer defense systems, several other classes of chemical defences are known in particular genera, and one of these is the triterpenoid saponins in the genus *Barbarea*^4^.

Within the crucifer family, several species and genera are used as model systems for evolution and chemical ecology of plant defense compounds, including *Arabidopsis*^5^, *Boechera*^6^,^7^, *Brassica* (cabbages)^8^, and *Barbarea*^9^,^10^,^11^,^12^. The *Barbarea* genus is especially interesting as it contains characteristic defense compounds: the saponins, which are unique in the crucifer family^9^,^13^,^14^, a range of rare or unique aromatic glucosinolates^2^,^15^, and newly discovered non-indole phytoalexins suggested to be glucosinolate derived^16^. Glucosinolates of *Barbarea* species are exclusively derived from phenylalanine and tryptophan. This is in contrast to glucosinolates from other crucifers, such as *A. thaliana, Boechera stricta* and cabbages, that also include glucosinolates derived from aliphatic amino acids. Triterpenoid saponins are glycosylated triterpenoids with soap-like physical properties, which serve multiple roles in pest and disease resistance^14^. Triterpenoids are common in crucifers, and it seems that the ability to produce saponins in the *Barbarea* species evolved by a novel substrate specificity of a newly duplicated UDP-glucosyl transferase^17^.

One of the species in the *Barbarea* genus, *B. vulgaris* R.Br., is additionally interesting because it includes two divergent ’types’ that differ in glucosinolate and saponin profile^15^,^18^,^19^. They also differ in their density of trichomes on rosette leaves; one is almost without trichomes (i.e. “glabrous”) and therefore called G-type, the other has high density of trichomes (“pubescent”) and is called P-type. Both types are diploid (2n = 2x = 16)^20^, with different, but overlapping, geographic ranges^18^.

The major G-type and P-type glucosinolates differ in the stereochemistry (either *S* or *R*, respectively) of hydroxylation of their common phenethylglucosinolate backbone, leading to epimeric glucobarbarins (Supplementary Fig. 1)^2^. Additional hydroxylation in the P-type leads to other P-type specific glucosinolates and hydrolysis products^2^. The biosynthetic pathway of glucobarbarins was recently proposed^21^. In general the P-type deviates markedly from the G-type and other investigated *Barbarea* species^19^, and is for this reason regarded as an ‘innovative’ evolutionary lineage with respect to specialized metabolites, including a number of rare and even unique glucosinolates and saponins^10^,^15^,^17^.

The five known saponins produced by the G-type of *B. vulgaris*, and the other *Barbarea* species tested so far, consists mainly of a mixture of different β-amyrin-derived saponins^10^,^17^. Notable among these are hederagenin cellobioside and oleanolic acid cellobioside. Especially the former is highly deterrent to some specialist lepidopteran herbivores, including the diamondback moth (*Plutella xylostella*), to the extent that the larvae will eventually die if no alternative host plant is available^4^,^22^. In contrast, P-type plants seem to produce mainly lupeol-derived saponins^17^, which are not known as deterrent or toxic to these specialist herbivores.

We previously detected QTLs for the biochemical differences between the G- and P-type in a population of F_2_ hybrids^10^. QTLs were detected for both G-type glucobarbarin (*S*-configuration) and P-type epi-glucobarbarin (*R*-configuration) on different linkage groups, clearly showing that different genes are involved. This is supported by recent transcriptomics analyses suggesting two related but quite diverged genes are responsible for the hydroxylations^21^. QTLs for G-type saponins have also been identified, together with genes involved in their biosynthesis^17^. However, to find additional genes and detect the evolutionary and functional changes that have diversified the plants and their defense metabolites, a genome of *B. vulgaris* was much wanted.

Here we report a draft genome sequence of the *B. vulgaris* G-type, and re-sequencing of the P-type. On the basis of a 168-Mb assembly we identify 25,350 protein coding genes, of which 81% are anchored to eight pseudomolecules. Comparative genomic analysis between the G- and P-types allow us to determine genetic differences between them, and using genetic analysis we propose candidate genes underlying their difference in trichome density and glucosinolates. The *B. vulgaris* genome will lead to a better understanding of the production of specialised metabolites conferring disease and insect resistance in general, and of evolutionary events leading to the loss of a particular insect resistance and changed glucosinolate profile and trichome density in the biochemically innovative P-type.

## Results

### Genome sequencing and assembly

We selected one outbred G-type individual for whole genome sequencing, from which we generated a total of 17.9 Gb of sequence data on the Illumina GAII system of two fragment libraries with different insert sizes. This represented approximately a 66.5 X coverage of the *B. vulgaris* genome, with an estimated size of 270 Mb based on k-mer spectrum analysis. These data were supplemented with a long jump distance library of 14.4 Kb in size, and 5.2 Gb of PacBio data (Supplementary Table 1). *De novo* assembly (Supplementary Fig. 2) of these sequences generated a draft genome assembly of 167.7 Mb, representing 62.1% of the estimated genome size (Table 1), when only taking contigs greater than 1000 bp into consideration. The remaining ~38% is likely consisting of repetitive regions that cannot be resolved using short read shotgun assembly. The assembly consists of 16,938 contigs and 7,874 scaffolds with N50 sizes of 14.3 Kb for contigs and 56.3 Kb for scaffolds (Table 1). Despite the smaller assembly size relative to the estimated genome size, the assembly provides a good representation of the gene space. This is demonstrated by the fact that 97% of 41,018 *de-novo* assembled transcripts from an RNAseq study^11^ had a valid alignment (Supplementary Table 2) in our assembly. Furthermore, we used a Core Eukaryotic Genes Mapping Approach (CEGMA)^23^ to evaluate the assembly for completeness, and it showed that 96% of core eukaryotic genes were present as complete hits and 98% were present as partial hits (Supplementary Table 3).

To determine scaffold placement on pseudomolecules we first attempted to anchor scaffolds by creating a high density genetic map of an F_2_ population derived from the selfing of an F_1_ plant from a cross between a heterozygous G-type and a P-type plant. Each F_2_ individual was genotyped by a genotype-by-sequencing (GBS) approach^24^ and we constructed a linkage map comprised of 796 markers spread across eight linkage groups (Supplementary Table 4 and Supplementary Fig. 3), which is in agreement with the chromosome number determined by cytogenetic analysis^20^. Using this map we could place 431 scaffolds into eight pseudomolecules, which had a total length of 38.7 Mb (23% of the assembly).

In a second strategy we used comparative analysis with the closely related genome of *Arabidopsis lyrata*^25^, to order and orientate the *B. vulgaris* scaffolds based on gene pairs in conserved synteny. The macro- and micro-synteny between *B. vulgaris* and *A. lyrata* has been evaluated (see material and methods), and there was good co-linearity between linkage groups and *A. lyrata* chromosomes, albeit with some re-arrangements within linkage groups. The *B. vulgaris* genetic map took precedence over synteny when ordering. The top of linkage group two had a segment (0–69.7 cM) that was linked to a segment of *A. lyrata* chromosome 6. A final pseudomolecule assembly was generated by integrating the anchoring information from the genetic map with that of the comparative map with *A. lyrata*. In total 122.1 Mb (72.8%) of the assembly was anchored to eight pseudomolecules, and 89.2 Mb (53.2%) was orientated.

### Gene prediction and functional annotation

We identified 25,350 protein-coding loci in the *B. vulgaris* genome using *de novo* and homology based gene predictions with the MAKER2^26^ pipeline. We assembled *de-novo* an available RNAseq data set^11^, and also used an *A. thaliana* protein set^27^ as evidence. Genes were found on 4,527 scaffolds with an average of 5.6 genes per scaffold. Using the GBS map, 7,525 genes (29.7%) were directly anchored to the genetic map, and 20,538 genes (81%) were anchored in the final assembly consisting of eight pseudomolecules. The average number of exons per gene was 6.1, and the average protein length was 415.7 amino acids (Supplementary Fig. 4), in agreement with metrics from *A. thaliana*^27^. Genes were assigned functional annotation using blastp searches (Supplementary Data 1). Of 25,350 predicted proteins, 20,006 (79%) had a blastp hit in the UniProt Viridiplantae sequences with an E-05 cut off. Furthermore, 24,826 (98%) predicted proteins had at least one predicted Pfam domain, 2,394 (9%) contained predicted signal peptides, and 5,301 (21%) transmembrane helices.

The 25,350 proteins of *B. vulgaris* were compared against proteins from *A. thaliana*^27^, *A. lyrata*^25^, *C. rubella*^28^, and *Brassica rapa*^29^ using the software OrthoMCL^30^. This revealed that 13,678 orthologous groups were shared among all five species, and only 162 were unique to *B. vulgaris* (Supplementary Fig. 5).

### Genetic diversity between *Barbarea vulgaris* chemotypes

To improve our understanding of genetic differences between the G- and P-type, we re-sequenced the P-type to complement the *de novo* G-type assembly. We identified 0.87 million and 1.26 million heterozygous variants in the G- and P-type plant, respectively, and 1.43 million variants that were homozygous for a different allele between G- and P-type individuals. The number of genes with heterozygous variants was 15,610 (62%) and 20,246 (80%) in G-type and P-type, respectively. The number of protein coding genes with fixed differences between the G-type and P-type was 22,555 (89%), and on average there were 29.6 fixed differences per gene. Fixed differences were well distributed along all eight pseudomolecules (Supplementary Fig. 6). Of the 1.43 million fixed differences, 79% were SNPs, 10% were insertions, and 11% were deletions, and these were well distributed across genomic features (Supplementary Fig. 7). A relatively large proportion of variants (9,266) were assigned to effect types considered to have a disruptive impact on a protein (Supplementary Fig. 8), making them candidate loci to explain phenotypic differences between plant types. These were distributed across 5,213 sequences associated with GO terms for metabolic processes, such as cellular aromatic compound metabolic process, cellular nitrogen compound metabolic process, and organic cyclic metabolic process (Supplementary Fig. 9). Considering the variation in saponins and glucosinolates between the G- and P-types, we searched for cytochromes P450 within the list of genes with fixed differences between the G- and P-types, and identified 42 sequences (Supplementary Data 2) with fixed differences likely to have a disruptive impact on protein function.

### *GL1* is a candidate locus differentiating trichome density

**Figure 1:**
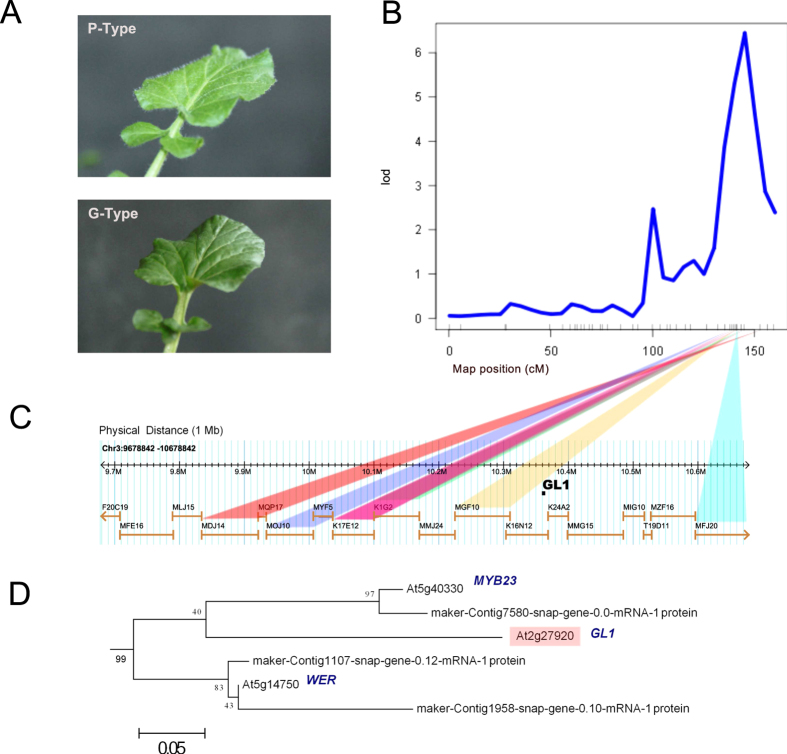
Differentiation in trichomes between P and G-type. (**A**) Phenotypic difference in trichome density between P and G type plants, (**B**) QTL on linkage group eight accounting for 29.8% of the phenotypic variation for trichome density, (**C**) comparative genomic analysis around the QTL region based on sequence homology between *B. vulgaris* scaffolds and *A. thaliana* BAC sequences, and (**D**) molecular phylogenetic analysis by the Maximum-Likelihood method using the JTT matrix-based model. The tree with the highest log likelihood is shown. Bootstrap values are shown next to the branches. The tree is mid-point rooted, drawn to scale, with branch lengths proportional to the number of substitutions per site. We used genes from the orthologus group containing the *A. thaliana* GL1, and genes from a BLASTP search using a coverage cutoff of 80% and a minimum identity threshold of 50%. The sub-clade containing GL1 is shown.

The trichomes found in *B. vulgaris* are simple and non-glandular^19^, and the two *B. vulgaris* genotypes are morphologically distinguished by and named from the scarcity of trichomes on rosette leaves in the G-type and abundance in the P-type (Fig. 1A). For this reason, it was an obvious first endeavor to use the genome for locating a candidate gene for this difference. Previous analysis of the F_2_ population described above identified two QTLs for pubescence; however, confidence intervals for these QTLs were large^10^. We used the newly developed GBS map to re-analyse the data on pubescence, and identified a QTL with large effect on linkage group eight, with a peak at 143.5 cM (Fig. 1B) and a 95% Bayesian confidence interval of 3.9 cM. Another QTL with smaller effect was identified on linkage group four, and taken together the two QTL model accounts for 34.1% of the phenotypic variance for trichome density (Supplementary Table 5). The QTL peak on linkage group eight is in a region with homology to a segment of *A. thaliana* chromosome three (Fig. 1C). A QTL for trichome density has already been identified in this region in an *A. thaliana* experimental mapping population^31^. The protein underlying this QTL is GL1 (AT3G27920), a MYB like transcription factor involved in activation of the developmental pathway for trichome differentiation^32^. Furthermore, GL1 has recently been shown to have qualitative and likely quantitative effects on trichome density in natural populations of *A. thaliana*^33^. GL1 is one of three proteins in the *A. thaliana* R2R3-MYB subgroup 15, together with MYB23 and WER^34^. We used GL1 as a query to search both *A. thaliana* and *B. vulgaris* proteins for similar sequences, and generated a phylogenetic tree. Not surprisingly, the three *A. thaliana* proteins GL1, MYB23, and WER were present in a sub-clade, together with three *B. vulgaris* proteins (Fig. 1D). MYB23 is functionally equivalent to GL1 with respect to trichome initiation but not branching. Two genes were located on scaffolds anchored to pseudomolecules 5 and 7, while the third gene (maker-Contig7580-snap-gene-0.0-mRNA1) was on an unanchored scaffold. This gene shares the greatest amino acid identity (gapped alignment) to GL1 (57.6%, Supplementary Fig. 10), and considering the other two genes are anchored outside the QTL region, is the most likely *B. vulgaris* ortholog to GL1. Our results suggest that an ortholog of *GL1* is a likely candidate gene explaining variation in trichome density between the glabrous (G) and pubescent (P) types of *B. vulgaris*.

### Genetic basis of contrasts to the Arabidopsis glucosinolate profile

**Figure 2:**
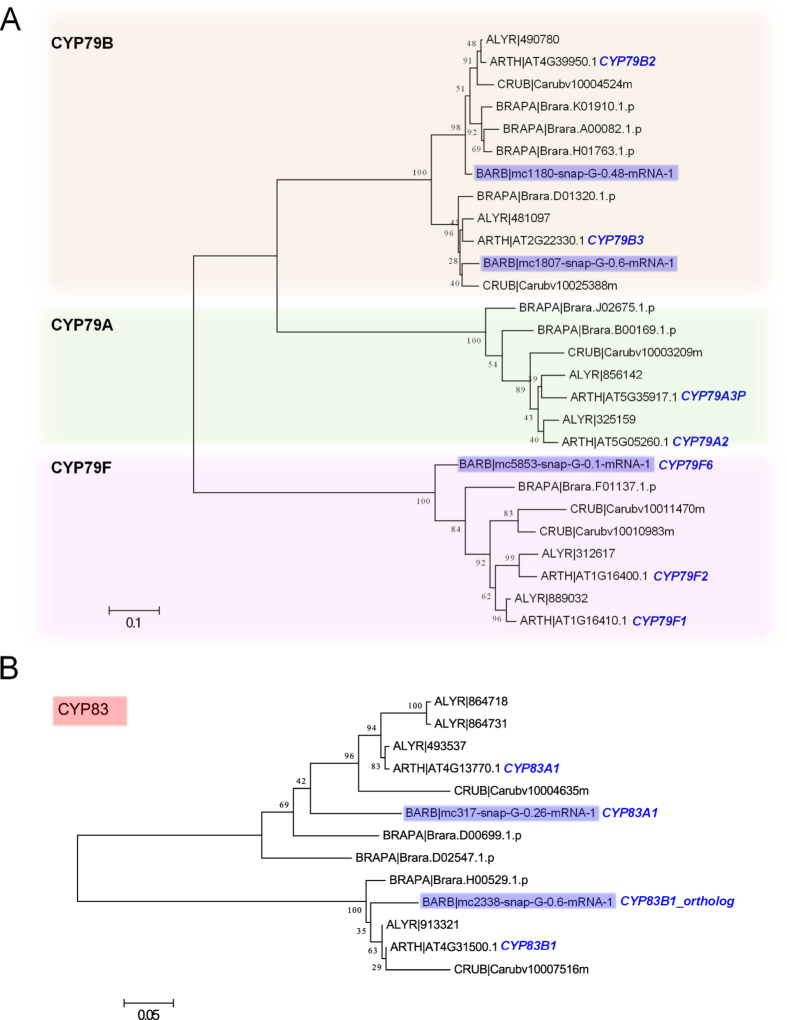
Phylogenetic analysis of CYP79F and CYP83 proteins. Molecular phylogenetic analysis by the Maximum-Likelihood method using the JTT matrix-based model. The tree with the highest log likelihood is shown. Bootstrap values are shown next to the branches. The tree is mid-point rooted, drawn to scale, with branch lengths proportional to the number of substitutions per site. We used genes from the orthologus group containing the *A. thaliana* CYP79F1 and F2 proteins (**A**), and proteins from the orthologus group containing the *A. thaliana* CYP83A1 and B1 proteins (**B**). *B. vulgaris* proteins are highlighted in blue, and *A. thaliana* proteins are labeled. See Supplementary Figs 11 and 12 for amino acid alignments.

A major contrast between glucosinolates in *A. thaliana* and the genus *Barbarea* is the apparent lack of methionine derived glucosinolates in *Barbarea*^15^,^19^. Comparison with close relatives of *Barbarea*^35^ suggests the lack of methionine derived glucosinolates is due to recent evolutionary loss. The entry of (chain elongated) methionine to glucosinolate biosynthesis in *A. thaliana* is controlled by the paralogous CYP79F1 and CYP79F2, while the genetic and enzymatic basis of the corresponding step for phenethylglucosinolate in *A. thaliana* is completely unknown^36^. We found only one *B. vulgaris* protein in an orthologous group with CYP79F1 and CYP79F2 (Fig. 2, alignments in Supplementary Fig. 11); the gene encodes an enzyme that is 82**%** identical to CYP79F1 and has been named CYP79F6 by the P450 nomenclature committee^21^. The gene is highly expressed and induced by diamondback moth infestation as expected for a gene responsible for biosynthesis of phenethylglucosinolate and derivatives such as glucobarbarins^21^. If CYP79F6 is responsible for the committed biosynthetic step to phenethylglucosinolate and glucobarbarins^21^, the apparent lack of methionine derived glucosinolates would seem to be due to a changed substrate specificity^37^ of CYP79F6. Our genome-wide search for homologues extends the previous transcriptome analysis of leaves^21^, and thereby supports the apparent key role of CYP79F6 in creating the difference between the *Barbarea* and *A. thaliana* glucosinolate profiles.

### Glucosinolate backbone biosynthesis proteins

We complemented the list of putative glucosinolate biosynthesis genes, known from the transcriptome^21^, by a genome-wide search. In *A. thaliana*, conversion of precursor amino acids to aldoximes by CYP79F genes are followed by oxidization to activated compounds by CYP83A1 in the aliphatic pathway. We identified putative orthologs to both CYP83A1, and CYP83B1 from the aliphatic, phenethyl and indole glucosinolate pathways (Fig. 2, alignments in Supplementary Fig. 12). We also identified a putative ortholog of GSTF11 (*BARB|mc650-snap-G-0.53-mRNA-1*) and SUR1 (*BARB|mc404-snap-G-0.41-mRNA-1,* Supplementary Fig. 9), which are involved in converting activated aldoximes to *S*-alkyl-thiohydroximates, and the subsequent conversion to thiohydroximates by SUR1. UGT74C1 is proposed to glucosylate methionine derived thiohydroximates to form aliphatic desulfoglucosinolates, and while we identified a putative ortholog of UGT74B1, which acts on the aromatic thiohydroximates, we did not identify an ortholog of UGT74C1 (Supplementary Fig. 13). The next step is the sulfation by sulfotransferases to form glucosinolates. SOT17 and SOT18 preferentially sulfate aliphatic substrates, and SOT16 Phe- and Trp- derived substrates. We identified putative orthologs to all three sulfotransferases (Supplementary Fig. 14), along with some closely related sulfotransferases that were only identified in *C. rubella, B. rapa*, and *B. vulgaris*.

### Aliphatic glucosinolate side chain decoration genes

**Figure 3:**
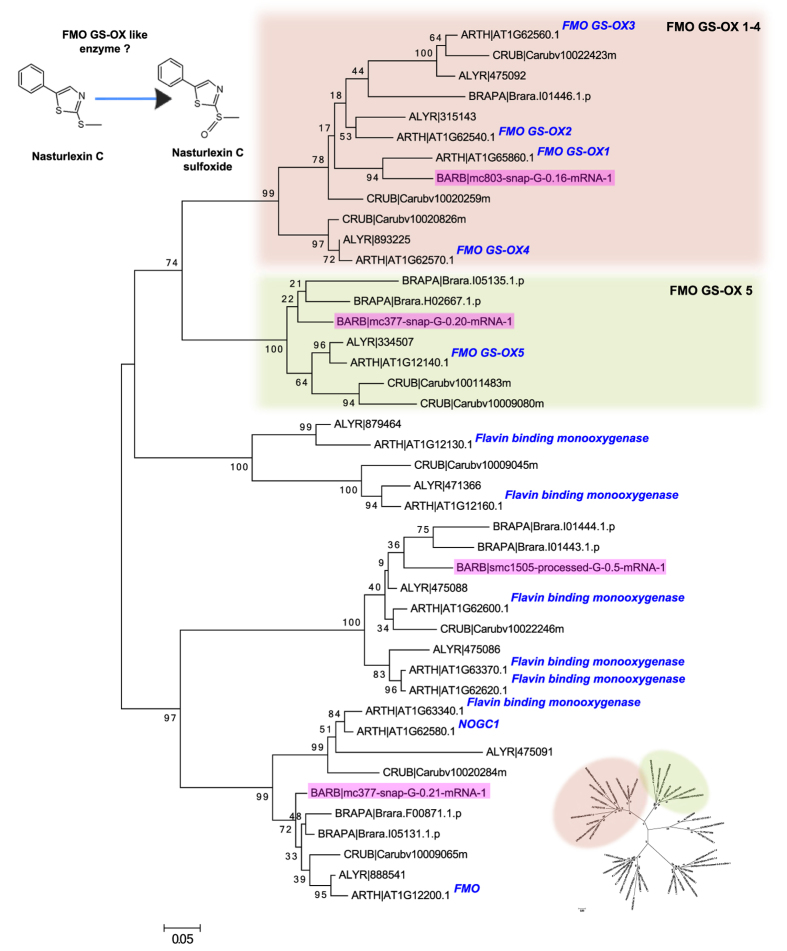
Phylogenetic analysis of FMO GS-OX proteins. Molecular phylogenetic analysis by the Maximum-Likelihood method using the JTT matrix-based model. The tree with the highest log likelihood is shown. Bootstrap values are shown next to the branches. The tree is mid-point rooted, drawn to scale, with branch lengths proportional to the number of substitutions per site. We analysed proteins from the orthologous group containing the *A. thaliana* FMO GS-OX proteins. *B. vulgaris* proteins are highlighted in blue, and *A. thaliana* proteins are labeled. Radial tree clearly showing the five distinct branches is shown in the bottom right. A suggested enzymatic function of one or more of the *B. vulgaris* FMO GS-OX proteins is indicated (top left).

Among the last steps of the biosynthesis of methionine derived glucosinolates in *A. thaliana* is the oxidation of methylthioalkyl glucosinolates to methylsulfinylalkyls by FMO-GSOX enzymes^36^. The finding of apparently two functional *FMO-GSOX* genes in *B. vulgaris* was initially surprising, since the standard substrates and products (methylthioalkyl and methylsulfinylalkyl glucosinolates) are apparently absent in the species^15^. There are five *FMO-GSOX* genes in *A. thaliana*, numbered 1–5, of which numbers 1–4 are biochemically similar and number 5 is slightly different in terms of substrate specificity^38^. Methylthioalkyl and methylsulfinylalkyl glucosinolates are known from close relatives of *Barbarea*^35^, and the common ancestor is expected to have had the *FMO-GSOX* gene. Phylogenetic analysis of genes clustering within an orthologus group containing FMO-GSOX proteins identified a sub-clade containing FMO-GSOX 1–4 from *A. thaliana* and a single protein from *B. vulgaris* (Fig. 3, alignments in Supplementary Fig. 15). Loss of *FMO-GSOX* genes fits expectations since *B. vulgaris* apparently lacks methionine derived glucosinolates.

An explanation for the continued existence of some *FMO-GSOX* genes in *B. vulgaris* could be that their biochemical function has changed. Indeed, apparently unique phytoalexins with either a methylthio group or a methylsulfinyl group were recently reported from *B. vulgaris*^16^ (Supplementary Fig. 1), and the identified *FMO-GSOX* genes may be involved in phytoalexin biosynthesis (Fig. 3). Comparing the four FMO-GSOX proteins with relevant sequences from other cruciferous species, we noticed two of the *B. vulgaris* proteins were placed in clades with one or more functionally characterized *A. thaliana* proteins involved in oxidation of thiomethyl groups in glucosinolates. However, the other two *B. vulgaris* “FMO-GSOX” proteins were placed in different clades, with *A. thaliana* proteins involved in oxidation-reduction. Apparently the four identified *B. vulgaris* genes represent considerable diversity, making them particularly interesting to investigate in a plant lacking the classical aliphatic glucosinolate substrates of these genes. Secondary modifications of aliphatic glucosinolates can also be achieved by AOP2 and AOP3, however, we didn’t identify any putative orthologs of AOP within the *B. vulgaris* assembly. Additional modifications are achieved by GS-OH, which is involved in hydroxylation, and *B. vulgaris* shows variation in hydroxylation between P- and G-types as described below.

### Genetic loci controlling glucosinolate side chain hydroxylation

**Figure 4:**
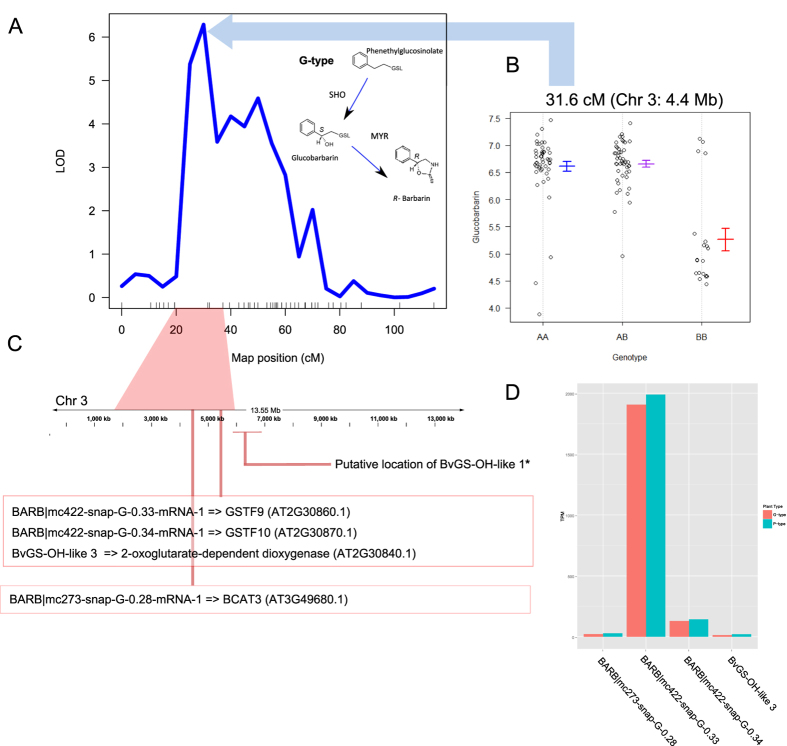
Genetic locus for variation in glucobarbarin content between G and P-type. A QTL on linkage group three accounting for 39.3% of the phenotypic variation for glucobarbarin (**A**), and a plot of the QTL effect at the SNP with the largest LOD score (**B**). The region spanning the QTL is linked to a position spanning approximately 4 Mb of *B. vulgaris* pseudomolecule three, within which four genes involved in glucosinolate biosynthesis have been anchored (**C**). The expression of these four genes (Transcripts Per Million), in both the P and G-type RNA-seq data is shown (**D**).

The G- and P-type glucosinolate profiles differ in the stereochemistry of 2-hydroxylation^15^. The resulting glucobarbarins have been indirectly linked to ‘dead-end’ resistance to the diamondback moth^9^,^39^, and to resistance to the cabbage moth^12^ and phytoalexin biosynthesis^16^. QTLs for variation in glucobarbarin ((2 *S*)-2-hydroxy-2-phenylethylglucosinolate) and epiglucobarbarin ((2 *R*)-2-hydroxy-2-phenylethylglucosinolate) were previously identified^10^, however, re-analysis with the GBS map has enabled the QTL be more precisely located. One QTL for glucobarbarin was identified on linkage group three accounting for 39.3% of the phenotypic variation, and one QTL for epiglucobarbarin was identified on linkage group four accounting for 53.1% of the phenotypic variation (Fig. 4, Supplementary Tables 6 and 7).

**Figure 5:**
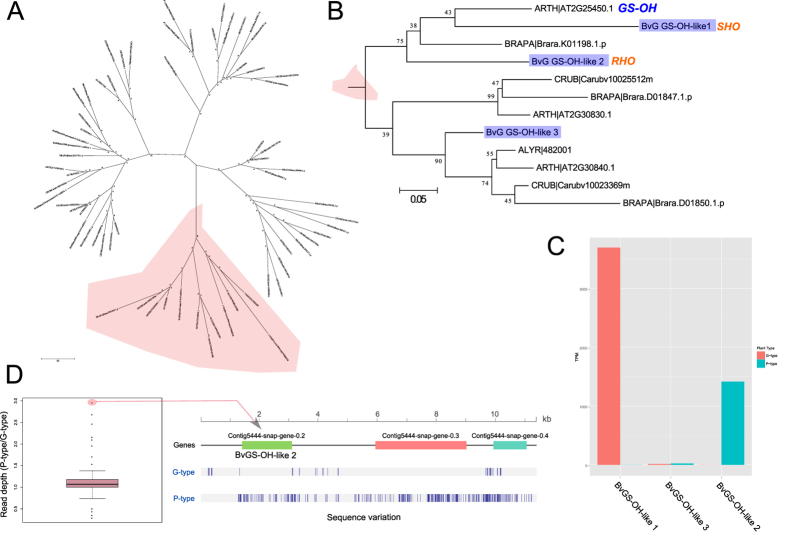
Phylogenetic analysis of homologs *to A. thaliana GS-OH* proteins. Molecular phylogenetic analysis by the Maximum-Likelihood method using the JTT matrix-based model. The tree with the highest log likelihood is shown. Bootstrap values are shown next to the branches. The tree is mid-point rooted, drawn to scale, with branch lengths proportional to the number of substitutions per site. We analysed proteins from the orthologous group containing the *A. thaliana GS-OH* protein, and proteins from a BLASTP search using a coverage cutoff of 80% and a minimum identity threshold of 50%. (**A**) Shows a radial tree clearly displaying four distinct branches, with the branch containing the *A. thaliana* protein GS-OH highlighted in red. A close up of this sub-clade is shown in (**B**), with *B. vulgaris* proteins highlighted in blue, and *A. thaliana* proteins labeled in blue. *B. vulgaris* G-type sequences were used, indicated by an added “G” in abbreviations: BvG GS-OH-like 1/2/3. *RHO* and *SHO* refer to nomenclature recently proposed for these genes^21^. The expression of these four genes (Transcripts Per Million), in both the P and G-type RNA-seq data is shown (**C**). The sequence variation around BARB|mc5444-snap-G-0.2-mRNA-1 (*SHO*) in G and P-type is shown in (**D**), together with a boxplot of the relative mean read depths between P and G types for 100 randomly selected genes and the gene BvG GS-OH-like 2, which is highlighted.

The 2-hydroxylation needed to form glucobarbarin from phenethylglucosinolate in *Barbarea* has a counterpart in *A. thaliana*, controlled by the *GS-OH* locus. It has already been shown in *A. thaliana* that the *GS-OH* locus is encoded by a 2-oxoacid-dependent dioxygenase (AT2G25450) that is required for the production of 2-hydroxybut-3-enylglucosinolate^40^. This results from oxidation of 3-butenylglucosinolate to generate either (2 *S*)-2-hydroxy-3-butenylglucosinolate (progoitrin) or the 2-epimer (epiprogoitrin). Using the *A. thaliana* GS-OH protein as a query we searched protein sets from *A. thaliana*^27^, *A. lyrata*^25^, *C. rubella*^28^, *B. rapa*^29^, and *B. vulgaris*, with minimum of 80% coverage and 50% identity. Phylogenetic analysis of the resulting proteins identified four sub-clades (Fig. 5), with one sub-clade containing AT2G25450 and two other *A. thaliana* proteins, one *A. lyrata* protein, three *B. rapa* proteins, and three *B. vulgaris* G-type proteins (Fig. 5). The three *B. vulgaris* proteins were provisionally named BvGS-OH-like 1 (*BARB|mc2865-snap-G-0.4-mRNA-1*), BvGS-OH-like 2 (*BARB|mc5444-snap-G-0.2-mRNA-1*), and BvGS-OH-like 3 (*BARB|mc422-snap-G-0.43-mRNA-1*).

Interestingly, *BvGS-OH-like 3* is found proximal to the QTL for glucobarbarin on linkage group 3 where the G-type allele is responsible for higher production of glucobarbarin, but the expression of *BvGS-OH-like 3* was low in both G- and P-types (Fig. 4). Three other glucosinolate-relevant genes were found nearby (Fig. 4), but their involvement in hydroxylation was excluded for biochemical reasons. BvGS-OH-like 1 and 2 were present on a sub-clade with the *A. thaliana* protein encoding the *GS-OH* locus. These corresponded to two sequences, referred to as *RHO* and *SHO,* proposed to underlie variation in epiglucobarbarin and glucobarbarin between P- and G-types in a recent transcriptome study^21^,^41^. *SHO* and *RHO* were identified as sequence homologs to GS-OH in the G- and P-type transcriptomes respectively, and showed low amino acid identity (68%) with each other^21^. It was thus proposed that they were two independent genes that diverged during separation of G- and P-types^21^. However, using the genome we were able to identify genes (*BARB|mc2865-snap-G-0.4-mRNA-1* and *BARB|mc5444-snap-G-0.2-mRNA-1*) that each had high sequence similarity (over 98%) to both BvGS-OH-like 1 (*SHO*) and BvGS-OH-like 2 (*RHO*) in the G-type assembly. Using the RNA-seq data it is obvious that the genes are expressed highly in either G- or P-type (Fig. 5) as previously observed for *SHO* and *RHO*^21^. In the case of *BvGS-OH-like 1 (SHO*) we do not find a homologous sequence in the P-type, based on both mapping P-type reads to the reference G-type assembly, and sequence searches of a *de-novo* P-type assembly. This gene may have been lost from the P-type during separation of the plant types, which is supported by the absence of any detectable expression of this gene in P-type (Fig. 5). The scaffold with this gene from G-type was not directly anchored within the pseudomolecule assembly. However, we identified *A. thaliana* orthologs to other genes on this scaffold and used genes up and downstream in the genome to fish for *B. vulgaris* orthologs that had been anchored. Assuming synteny within this region, the likely location of this scaffold is between 5.9 and 6.9 Mb on chromosome three, placing it close to the QTL for glucobarbarin (Fig. 4). This evidence suggests that the very reduced levels of glucobarbarin in the P-type, compared to the G-type, could be due to loss of the *BvGS-OH-like 1 (SHO)* gene. Conversely, the high levels of glucobarbarin in the G-type could be due to very high expression of *BvGS-OH-like 1* in G-type leaves.

The G-type allele of *BvGS-OH-like 2* shared more than 98% identity with the *RHO* transcript identified in the P-type transcriptome by Liu *et al*.^21^. Although the gene is present in both types its expression is very different, with transcript accumulation only detected in the P-type plant (Fig. 5). When we inspect the sequence variation for this gene in both types, we see that the gene is completely homozygous in the G-type and appears highly heterozygous in the P-type (Fig. 5), however, read depth analysis suggests this gene is duplicated in the P-type plant (Fig. 5). *BvGS-OH-like 2* was located on an unanchored 11.37 Kb scaffold, and we found no sequence homologous to this scaffold in the *A. thaliana* genome. Of the three genes predicted within this scaffold, only *BARB|mc5444-snap-G-0.2-mRNA-1 (RHO*) had a significant match to a *A. thaliana* gene. This was *GS-OH*, although we know from the phylogenetic analysis that the GS-OH protein is more likely to be orthologous to *BARB|mc2865-snap-G-0.4-mRNA-1 (SHO*) (Fig. 5). Based on this, it appears that there are no sequences that are homologous to this region in *A. thaliana*. We went back to the GBS marker data, before applying filters based on segregation distortion and missing rate, in order to identify a marker located within this scaffold. We identified one marker with data missing for 40/111 individuals, and displaying segregation distortion (chi-squared equal to 17.28). However, when including this marker in linkage mapping it grouped with linkage group four and had maximum linkages with two makers just downstream of the QTL location for epiglucobarbarin on linkage group 4 (Supplementary Fig. 16). This QTL accounts for 53.1% of the phenotypic variation for epiglucobarbarin (Supplementary Table 7), and the evidence suggests that a *BvGS-OH-like 2 (RHO*) allele in the P-type plant is responsible for its accumulation.

### Insect resistance and saponins

QTLs for insect resistance have previously been identified and found to co-locate with QTLs for saponin content, and the OSCs (oxidosqualene cyclase) *LUP2* and *LUP5* genes^10^,^17^. In that study, the two QTLs were placed on separate linkage groups; however, in the improved analysis presented here they are located on a single linkage group (Supplementary Fig. 17). *LUP5* could be directly found in the assembly, and *LUP2* was anchored to the genetic map using a previously designed molecular marker. The QTL with the largest effect on resistance was located on linkage group 4 proximal to *LUP5*, and a QTL with smaller effect was also located on linkage group 4 proximal to *LUP2* (Supplementary Fig. 17). The resistance QTL proximal to *LUP5* co-located with QTL that have a large effect on the content of four known G-type saponins: hederagenin cellobioside, oleanolic acid cellobioside, gypsogenin cellobioside, and 4-epihederagenin cellobioside. These saponins have been shown to accumulate upon insect and pathogen attack^42^.

Our genotyping by sequencing (GBS) analyses greatly improved the previously published genetic map of *B. vulgaris*, and narrowed the genomic regions containing QTLs for insect resistance and saponins. As previously shown, our current analysis supports that the triterpenoid and glucosinolate pathways are unlinked, as the genes are not clusterered as has been shown with other pathways for plant specialized metatbolites^43^. Key enzymes involved in the biosynthesis of saponins in *B. vulgaris* were recently identified^17^, however the key gene involved in catalysing the C23 hydroxylation to hederagenin, the important insecticidal saponin, remains a mystery. Our present genome sequence and improved genetic map will stimulate future research into the tritepenoid pathway to fully elucidate the genes involved in biosynthesis of saponins and how they have evolved.

## Discussion

We have sequenced the genome of *B. vulgaris* using a combination of Illumina paired-end sequencing data and PacBio long reads. The resulting assembly is 167.8-Mb and covers 62.1% of the estimated genome size; however, it is estimated to provide a near full coverage of the gene space. The assembly consists of 25,350 protein coding genes, and we have used a combination of genetic linkage mapping and synteny with *A. lyrata* to anchor 72.8% of the assembly to eight pseudomolecules. The availability of the *B. vulgaris* genome provides a valuable genomic resource to study the production of rare or unique metabolites with ecological effects. As the first species to be sequenced within the genus *Barbarea*, it also adds a valuable resource for comparative genomics and evolutionary analysis within the crucifer family.

Two divergent types of *B. vulgaris*, G and P, can be distinguished based on the presence or absence of simple trichomes^19^. Trichomes have no known ecological effect in this species, but well known effects in other plants^44^. Loci controlling trichome density were previously mapped, but the genes underlying them have not been identified. Here, we developed a high density genetic linkage map and were able to more precisely map a major locus affecting trichome density to a small region on linkage group eight. The QTL region was syntenic with a region in *A. thaliana* containing the *GL1* locus, which is required for induction of trichome development^32^. In *A. thaliana*, the *GL1* locus is an important source of natural variation in trichome density^33^. Our results suggest that an ortholog of *GL1* is a likely candidate gene to explain much of the variation in trichome density that we observe between the glabrous and pubescent types of *B. vulgaris*.

Apart from trichome density, the two chemotypes differ in the types and relative abundances of glucosinolates they produce. While the parent glucosinolates are the same, tryptophan derived indol-3-ylmethylglucosinolate and homophenylalanine derived phenethylglucosinolate, the substitution patterns differ in multiple ways, with known or expected effects in the bioactive down-stream hydrolysis products^1^,^15^. As these interesting structures have not been identified in *A. thaliana* and crop plants, they are candidates for new resistance properties^2^,^10^,^16^,^21^. With the availability of genomic data, the two types of *B. vulgaris* provide an excellent model system for identifying the underlying genetics and biochemistry and exploring ecological effects. The gene classes selected here, potentially involved in stereospecific glucosinolate hydroxylation as well as phytoalexin biosynthesis, serve as examples of the biochemistries that can be explored in this model system.

The biosynthesis of glucobarbarin and epiglucobarbarin is hypothesised to result from hydroxylation of the common precursor 2-phenylethylglucosinolate^10^. Their relative abundancies vary in different tissues^2^,^19^, but usually glucobarbarin is most abundant in the G-type and epiglucobarbarin in the P-type^15^. Three genes were discovered, provisionally numbered 1, 2 and 3, that could be potentially involved in this difference, all sequence homologs of *GS-OH* in *A. thaliana*. Two of these, alternatively named *RHO* and *SHO*^15^,^21^, show extremely high expression in leaves. We propose that *BvGS-OH-like 2 (RHO*) controls epiglucobarbarin production. In the analysed G-type plant, the gene is completely homozygous and does not appear to be transcribed to detectable levels. In contrast, the gene appears to be duplicated in the P-type plant and is transcribed to a very high level. We propose that *BvGS-OH-like 1 (SHO*) is involved in glucobarbarin production in the G-type, but appears to be lost from the P-type; this is supported by the transcriptome data of Liu *et al*.^21^. Our discovery of *BvGS-OH-like 2 (RHO*) also in the G-type, and of a third homolog, *BvGS-OH-like 3* in both types, paves to way for studies of the evolution of glucosinolate decoration in the genus, leading to the aberrant P-type profile. Future studies should focus on biochemical and sequence variation in already established panels of diverse *B. vulgaris* genotypes^12^,^15^ to correlate sequence variation and glucosinolate decoration, and test the proposed roles of these genes in glucosinolate decoration with functional approaches such as those descibed in Khakimov *et al*., (2015)^45^.

The *B. vulgaris* draft genome sequence will be an important resource for studying defense compounds such as saponins, glucosinolates and phytoalexins. We augmented the G-type sequence by resequencing the P-type, which produces different structures of these defense compound classes with different bioactivities. A greater understanding of genes involved in the biosynthesis of novel glucosinolates, phytoalexins and saponins may enable breeding of crops with enhanced defenses against diseases and herbivorous pests.

## Material and Methods

### DNA preparation and whole genome shotgun sequencing

High quality genomic DNA was isolated from leaves of a G-type *B. vulgaris* individual using Qiagen kits (DNeasy Plant kit and Genomic-tip). Illumina paired-end (PE) libraries with mean fragment lengths of 130 and 500 bp were prepared from genomic DNA and sequenced. Long Jump Distance (LJD) libraries with average insert sizes of 17 Kb were prepared for the G-type and sequenced on an Illumina HiSeq 2000 by Eurofins Genomics (Ebersberg, Germany). For PacBio sequencing DNA from the G-type were prepared for sequencing (C2 chemistry), which was carried out at the Genome Sequencing and Analysis Core Resource at Duke University, NC, USA. The sequencing effort for each library varied (Supplementary Table 1).

### Genome assembly and annotation

The G-type was assembled as follows (Supplementary Fig. 2): the insert size of the short fragment library was less than twice the read length, therefore the reads were error-corrected and the pairs merged using the stand alone error-correcting (and fragment filling) algorithm in ALLPATHS-LG^46^. The Illumina data from fragment libraries (merged reads and 500 bp PE libraries) were assembled using Celera Assembler^47^ and scaffolding using the PacBio data was performed with SSPACE-LONG^48^. Long range information provided by Long Jump Distance (LJD) libraries (Eurofins, Germany), were used for scaffolding with SSPACE^49^. We then attempted to fill gaps in the assembly using the PacBio reads with PBJelly^50^. Annotation was performed with the MAKER2 annotation pipeline^26^, using *B. vulgaris* transcript data and *A. thaliana* proteins^27^ as initial evidence. The transcript evidence was generated by performing a *de-novo* assembly of publicly available RNA-seq data from a G-type *B. vulgaris* genotype^11^ using Trinity^51^. Genes were initially predicted directly from evidence, and a training file for SNAP^52^ was created. *Ab-initio* predictions were then generated by SNAP, and an updated training file developed. A further four iterations of gene prediction followed by an updating of the training file were completed. Genes were assigned functional annotation using Blastp searches against a database containing all UniProt Viridiplantae sequences (retrieved 08-02-2015) and the top hit was recorded (Supplementary Data 1). HMMER v.2.3^53^, SIGNALP v.4.1^54^, and TMHMM v.2.0^55^ were further employed to identify specific protein domains, signal peptides, and transmembrane helices.

### Evaluation of genome completeness (gene content)

We used CEGMA^23^ to evaluate the completeness of the assembly based on the conservation of 248 core eukaryotic genes. We also aligned the *de-novo* assembled *B. vulgaris* transcripts (41, 018) described above to the assembly using BLAT^56^. The results were parsed^57^ to identity the number of transcripts with a match in the assembly, the base coverage, and how the proportion of transcripts split across multiple scaffolds.

### Genotyping F2 population and genetic linkage mapping

We used an existing F_2_ mapping population that had previously been developed in our group by selfing an F_1_ plant from a cross between a G-type and P-type plant. *B. vulgaris* is highly outcrossing and the parental plants therefore are not fully homozygous. As the segregating F_2_ population was derived from a single F_1_ plant, all co-dominant markers are therefore expected to segregate 1:2:1. We genotyped the parents, F_1_ hybrid and 111 individuals of the F_2_ population. Genotyping was performed using a genotyping-by-sequencing protocol as described by Elshire *et al*.^24^. DNA was quantified using the Quant-iT Assay (Life Technologies), and 100ng of DNA was digested with PstI and ligated to modified Illumina adaptors containing the restriction site overhang and a unique bar-code sequence of between four and nine nucleotides. Two libraries were prepared and each was sequenced on four lanes of an Illumina HiSeq2000. This was done to reduce the amount of missing data and increase read-depth to improve our ability to call heterozygotes. Adaptor contamination was removed using Scythe (https://github.com/vsbuffalo/scythe) with a prior contamination rate set to 0.40. Sickle (https://github.com/najoshi/sickle) was used to trim reads when the average quality score in a sliding window (of 20 bp) fell below a phred score of 20. At this point reads shorter than 40 bp were also discarded. The reads were demultiplexed using sabre (https://github.com/najoshi/sabre), and all reads originating from the same sample were combined. Reads were aligned to the draft *B. vulgaris* assembly using BWA^58^, and the Genome Analysis Tool Kit (GATK)^59^ was used to generate a list of putative SNPs. We filtered out positions with a mapping quality below a phred score of 30, and only called genotypes with a genotype-quality-phred score of at least 30. Genotype calls with a phred score below 30 were assigned as missing values. We then filtered out all sites that were not heterozygous in the F1, and sites that had more than 50% of individuals with missing genotype calls. Any positions that were not heterozygous in the F1 were removed from further analysis. Genotypes homozygous for the reference allele (G-type genome) were identified as coming from the G-type parent, and genotypes homozygous for the variant allele were identified as coming from the P-type parent. Over 62% of markers heterozygous in the F1 were homozygous in both of the parents, and over 80% were homozygous in one parent. Genetic linkage mapping was carried out using JoinMap 4.1^60^,^61^. Severely distorted or monomorphic markers were removed before grouping into linkage groups with a minimum LOD score of 10. We identified suspect linkages when the recombination fraction was larger than 0.5. Mapping within each linkage group was achieved using the regression mapping algorithm. (Supplementary Fig. 2). This map was used to anchor the genome assembly. A subset of 355 markers well distributed across eight linkage groups were selected and used to generate a less redundant genetic map for QTL mapping (Supplementary Fig. 18). R/QTL was used to generate a plot of recombination fraction and LOD score along each linkage group (Supplementary Fig. 19). With this plot we can identify regions where we may have incorrectly encoded the parental alleles. The plot looks as expected, in that we see low-recombination fractions and high LOD scores along the diagonal.

### Comparative gene analysis

OrthoMCL^30^ was carried out to identify orthologous groups of genes using proteins from *A. thaliana*^27^, *A. lyrata*^25^, *C. rubella*^28^, *B. rapa*^29^, and *B. vulgaris.* All-vs-all BLASTP with a cut-off value of 10e-05 was used to identify putative orthologs based on reciprocal best similarity pairs. The MCL algorithm is then applied to a similarity matrix with an inflation value (-I) of 1.5. This results in groups with orthologous genes across species, and “recent” paralogs within species. Phylogenetic trees were generated in MEGA6^62^ using the Maximum Likelihood method based on the JJT matrix-based model^63^. All positions containing gaps and missing data were eliminated, and trees were drawn to scale, with branch lengths measured in the number of substitutions per site.

### Genome anchoring

We anchored the genome into eight pseudomolecules with the aid of the genetic linkage map and synteny with the *A. lyrata* genome^25^. Markers on the genetic linkage map are already linked to genomic scaffolds as a consequence of using the draft sequence as a reference for SNP discovery. In order to take advantage of synteny with *A. lyrata* we identified gene pairs between B. vulgaris gene predictions and those from *A. lyrata.* To do this we only selected 1:1 orthologs from an OrthoMCL analysis between proteins from the two species. We identified gene pairs that we were able to use for anchoring. The final pseudomolecule assembly was generated from the genetic map and synteny evidence using ALLMAPS^64^, where higher weighting was given to evidence from the genetic map. The macrosynteny between *B. vulgaris* and *A. lyrata* was compared by anchoring the genetic markers to the *A. lyrata* genome using gene-pairs identified with the OrthoMCL analysis (linking a gene in a mapped contig with the physical position of its putative ortholog in *A. lyrata*). Comparisons for each linkage group are shown in Supplementary Figs 20–27. Images were generated using AutoGRAPH^65^. We also evaluated the microsynteny between *B. vulgaris* and *A. lyrata* using SimpleSynteny^66^ for several *B. vulgaris* contigs (Supplementary Figs 28–30). *B. vulgaris* contigs with sequence homology to *A. lyrata* chromosome 1 were selected and genes within these were used in BLAST searches against the complete sequence of *A. lyrata* chromosome 1 using a minimum evalue of 0.0001 and a minimum coverage cutoff of 40%. Annotations were lifted from the scaffolds onto the pseudomolecule assembly and can be visualized at http://plen.ku.dk/Barbarea.

### QTL Analysis

QTL analysis for the traits analysed here was previously carried out in this population using a linkage map developed on a limited set of SSR markers, and dominant AFLP markers^10^. We used the less redundant genetic map (355 markers) for QTL analysis in R/QTL^67^,^68^ together with phenotype data for glucosinolates, hairs, and resistance already available^10^. A LOD threshold was calculated for each trait with 1000 permutations, and served as the threshold above which QTL were identified using the scanone function. This was used as a starting model for multiple-QTL modeling. An initial QTL object was created with the function makeqtl, followed by refinement with refineqtl. The QTL object was fitted with fitqtl, and we searched for evidence of additional QTL with addqtl. In the case of evidence for an additional QTL, a new QTL model was built and the process repeated. All calculations and plots were generated within the R environment^69^.

### Re-sequencing of a *B. vulgaris* P-type plant and variant analysis

High quality genomic DNA was isolated from leaves of a P-type *B. vulgaris* individual using a DNeasy Plant Kit (Qiagen). An Illumina paired-end (PE) library with a mean fragment length of 316 bp was prepared from genomic DNA and sequenced on an Illumina HiSeq2000 as paired end libraries with a 100 cycles. Reads were aligned to the draft G-type reference genome using BWA^58^, and duplicates marked using Picard Tools (http://broadinstitute.github.io/picard). The Genome Analysis Tool Kit (GATK) was used to generate a list of putative INDELS and perform re-alignments around these regions, and call putative INDELS and SNPs^59^. In addition to aligning P-type reads we also aligned reads from the G-type. We called genotypes when the genotype quality score was at least 30 (Phred scale), and filtered for positions that were heterozygous in either P or G-type genomes, or represented fixed differences between both types. Variant annotation was performed with SNPeff^70^, making use of the genome annotation to predict SNP effect types.

### Data Availability

This Whole Genome Shotgun project has been deposited at DDBJ/ENA/GenBank under the accession LXTM00000000. The version described in this paper is version LXTM01000000. Sequence variation between G- and P-type plants, and annotations, are available as tracks in the *Barbarea vulgaris* Genome Database, http://plen.ku.dk/Barbarea. Data referenced in this study are available in NCBI with the accession codes SRR1582492^11^ and SRR1583630^41^.

## Additional Information

**How to cite this article:** Byrne, S. L. *et al*. The genome sequence of *Barbarea vulgaris* facilitates the study of ecological biochemistry. *Sci. Rep.* **7**, 40728; doi: 10.1038/srep40728 (2017).

**Publisher's note:** Springer Nature remains neutral with regard to jurisdictional claims in published maps and institutional affiliations.

## Supplementary Material

Supplementary Figures and Tables

Supplementary Dataset 1

Supplementary Dataset 2

## Figures and Tables

**Table 1:** Summary statistics of *Barbarea vulgaris* genome assembly.

	Contigs (>1 Kb)	Scaffolds (>1 Kb)
Sum (Mb)		167.7
Total Number	16,938	7,874
N50 (bp)	14,305	56,364
Mean (bp)	8,989	21,304
Max (bp)	165,653	521,601
Captured Gaps (Mb)		15.4
Number in 8 Pseudomolecules		2,252
Sum (Mb) in 8 Pseudomolecules		113.2
