# Supplementary material for: The genome sequence of Barbarea vulgaris facilitates the study of ecological biochemistry
Source: Sci Rep. 2017 Jan 17;7:40728. doi: 10.1038/srep40728 (PMC5240624; doi:10.1038/srep40728)
Supplement: Supplementary Figures and Tables [file srep40728-s1.pdf]

The genome sequence of *Barbarea vulgaris* facilitates the study of ecological biochemistry

Stephen L. Byrne[1,2], Pernille Østerbye Erthmann[3], Niels Agerbirk[3], Søren Bak[3*], Thure Pavlo Hauser[3], Istvan Nagy[1], Cristiana Paina[1], Torben Asp[1*].

[1]Department of Molecular Biology and Genetics, Aarhus University, Forsøgsvej 1, 4200 Slagelse, Denmark.

[2]Crops, Environment & Land Use Programme, Teagasc, Oak Park, Ireland.

[3]Department of Plant and Environmental Sciences and Copenhagen Plant Science Center, University of Copenhagen, Thorvaldsensvej 40, 1871 Frederiksberg C, Denmark.

*Correspondence to: Torben Asp (torben.asp@mbg.au.dk), and Søren Bak (bak@plen.ku.dk).

Supplementary Figures

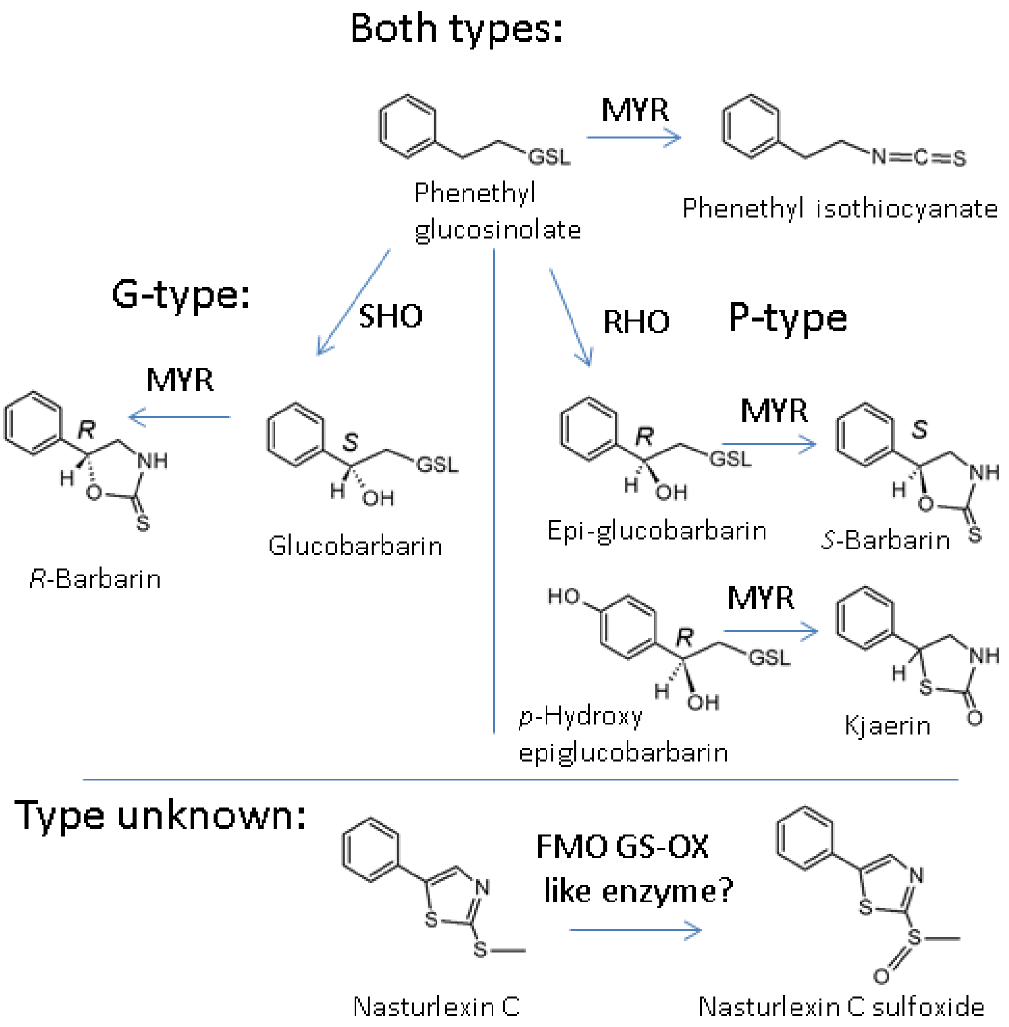

Supplementary Figure 1: Glucosinolate biosynthesis in B. vulgaris. Overview of glucosinolate hydroxylation in P-type and G-type.

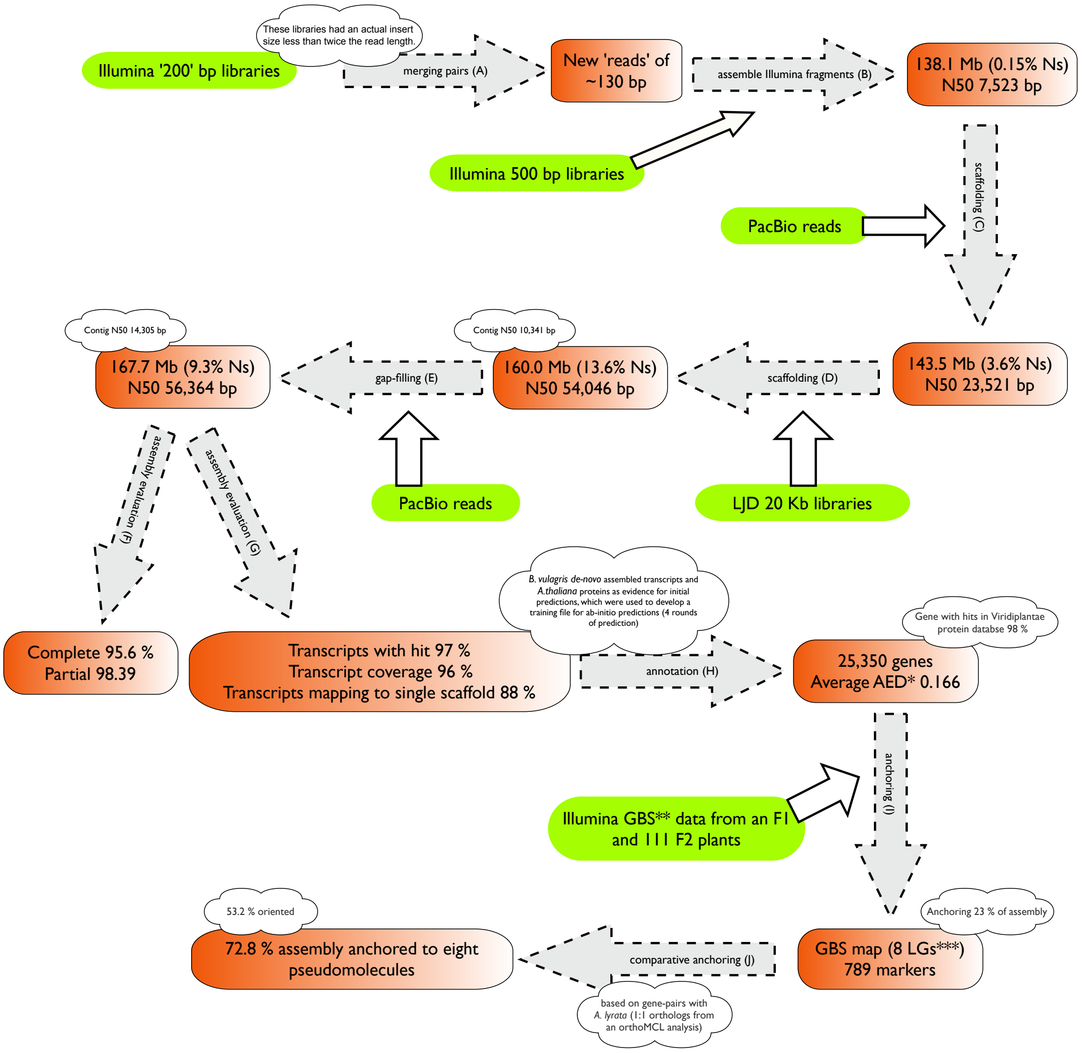

Supplementary Figure 2: Assembly workflow. Overview of assembly workflow. (A) merging pairs using the stand alone error-correcting (and fragment filling) algortihtm in ALLPATHS-LG, (B) Illumina assembly with Celera Assembler, (C) inter contig scaffolding with PacBio data using SSPACE-LONG, (D) scaffolding with LJD libraries using SSPACE, (E) intra-scaffold gapfilling with PacBio reads using PBJelly, (F) evaluation of gene content with CEGMA, (G) alignments of transcripts to genome with blast, (H) annotation with Maker2, (I) genotyping-by-sequencing genetic linkage map generated in JoinMap, (J) synteny based anchoring with ALLMAPS and gene pairs identified using OrthoMCL.

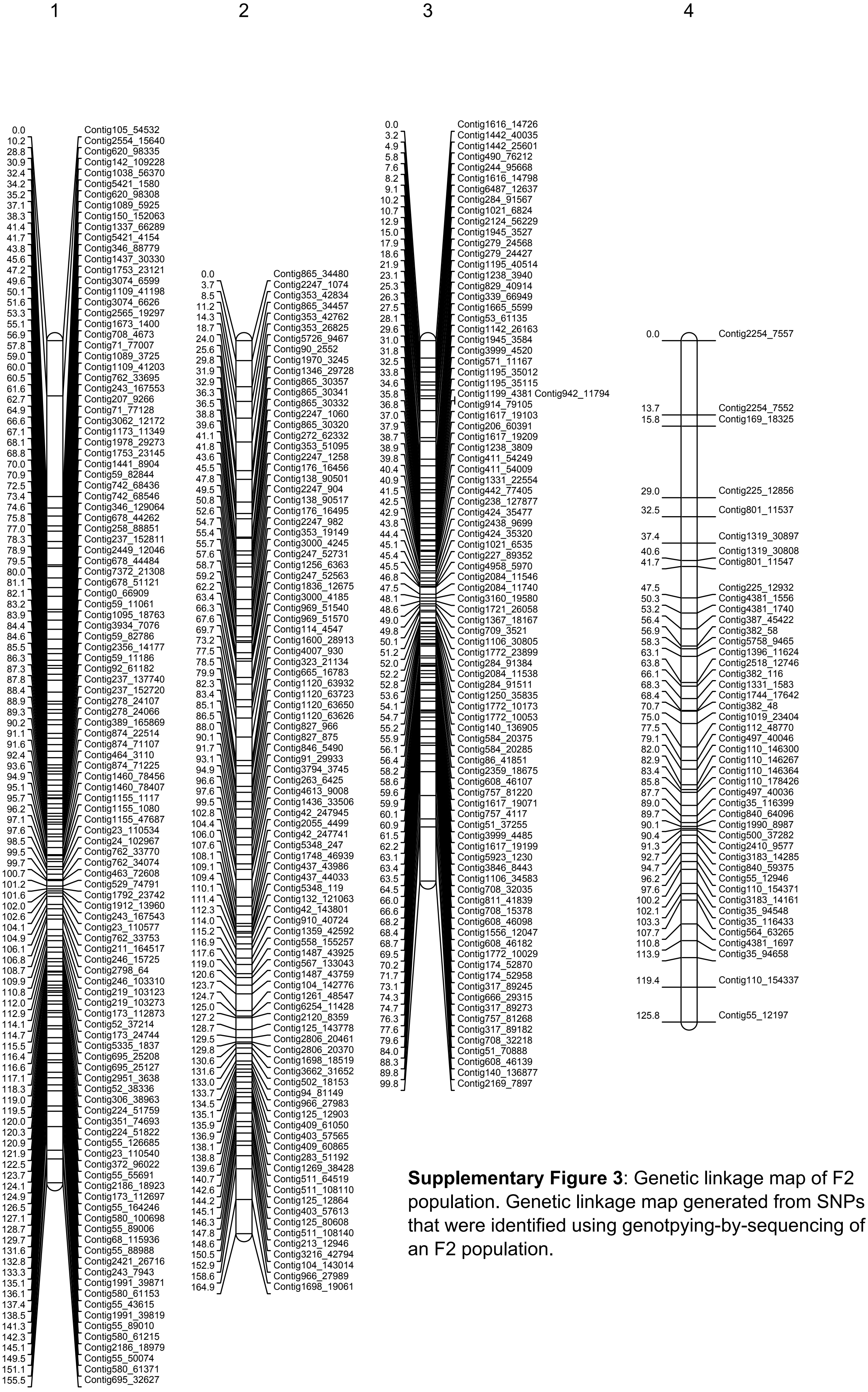

Supplementary Figure 3: Genetic linkage map of F2 population. Genetic linkage map generated from SNPs that were identified using genotpying-by-sequencing of an F2 population.

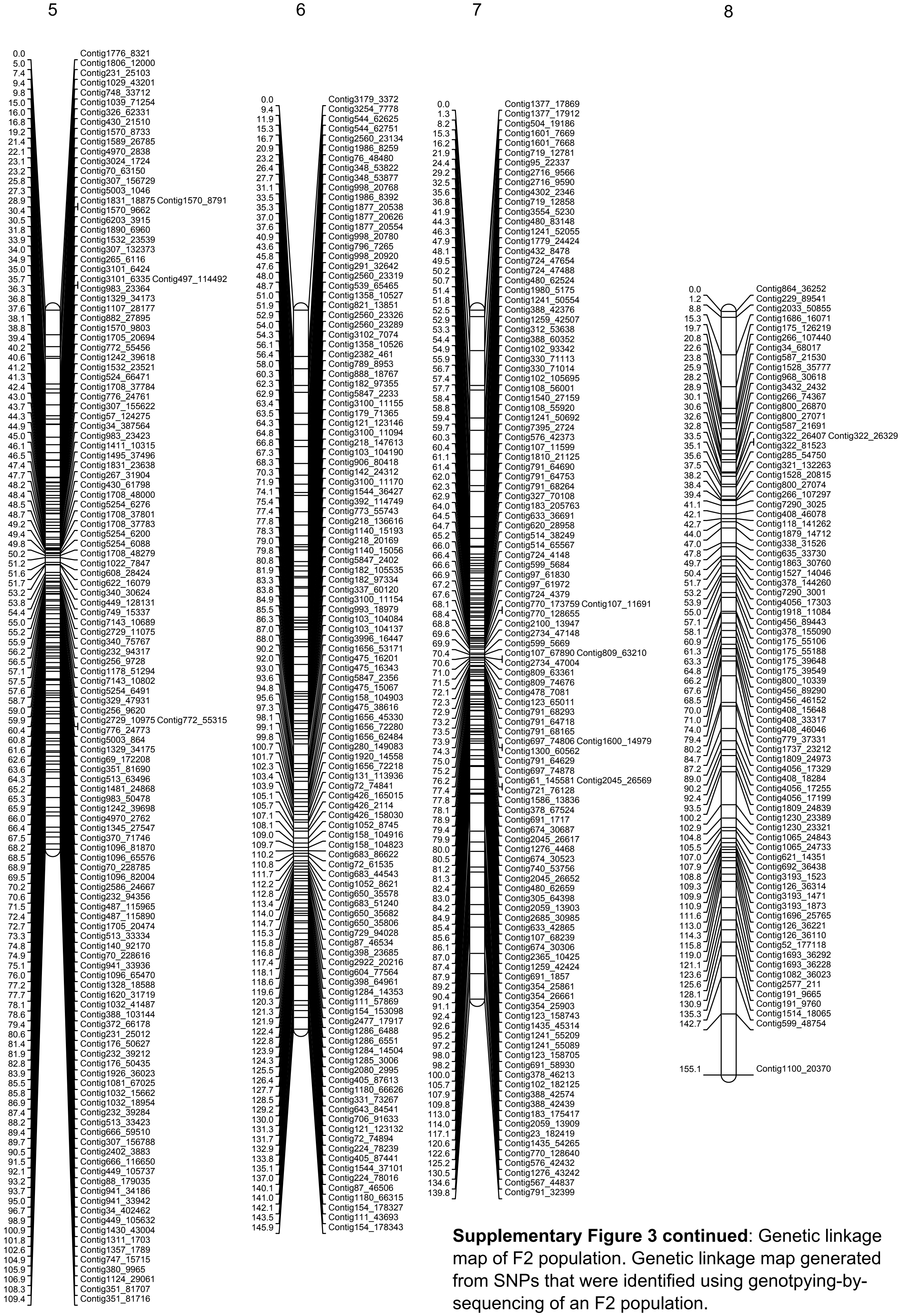

Supplementary Figure 3 continued: Genetic linkage map of F2 population. Genetic linkage map generated from SNPs that were identified using genotpying-by-sequencing of an F2 population.

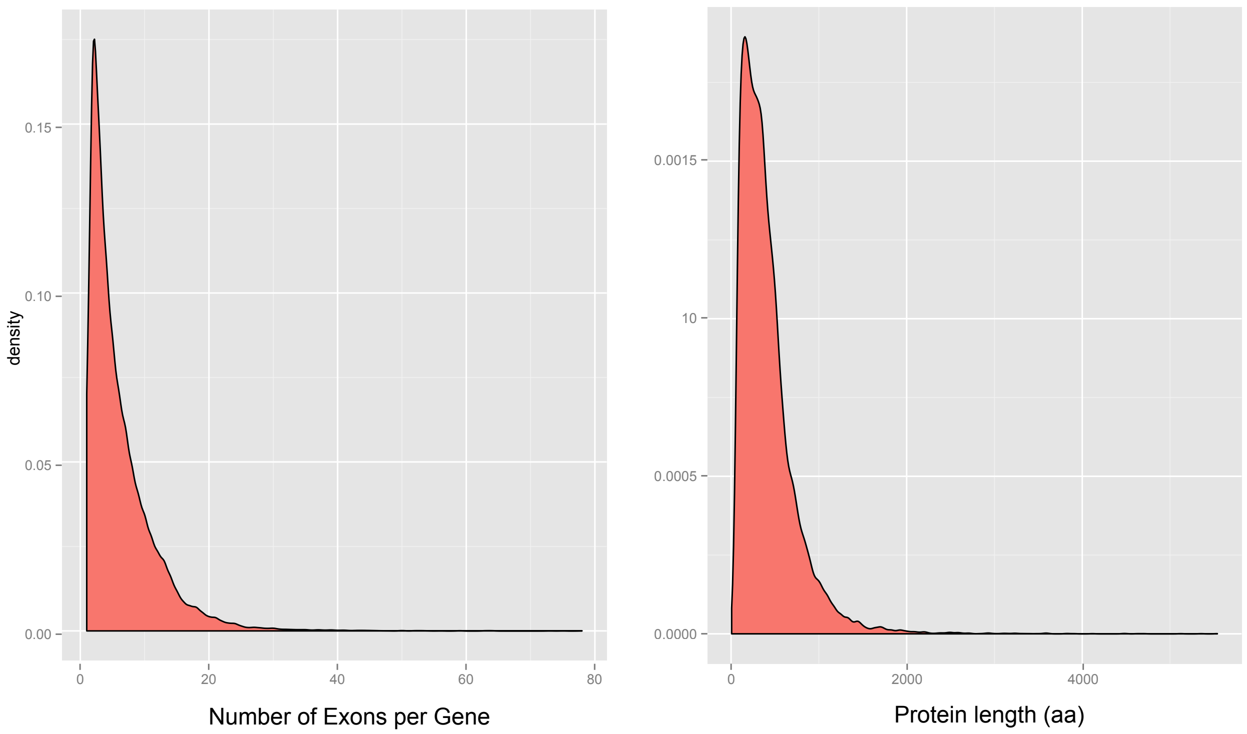

Supplementary Figure 4: Gene statistics. Density plots of the number of exons per gene (left), and the length of proteins (right).

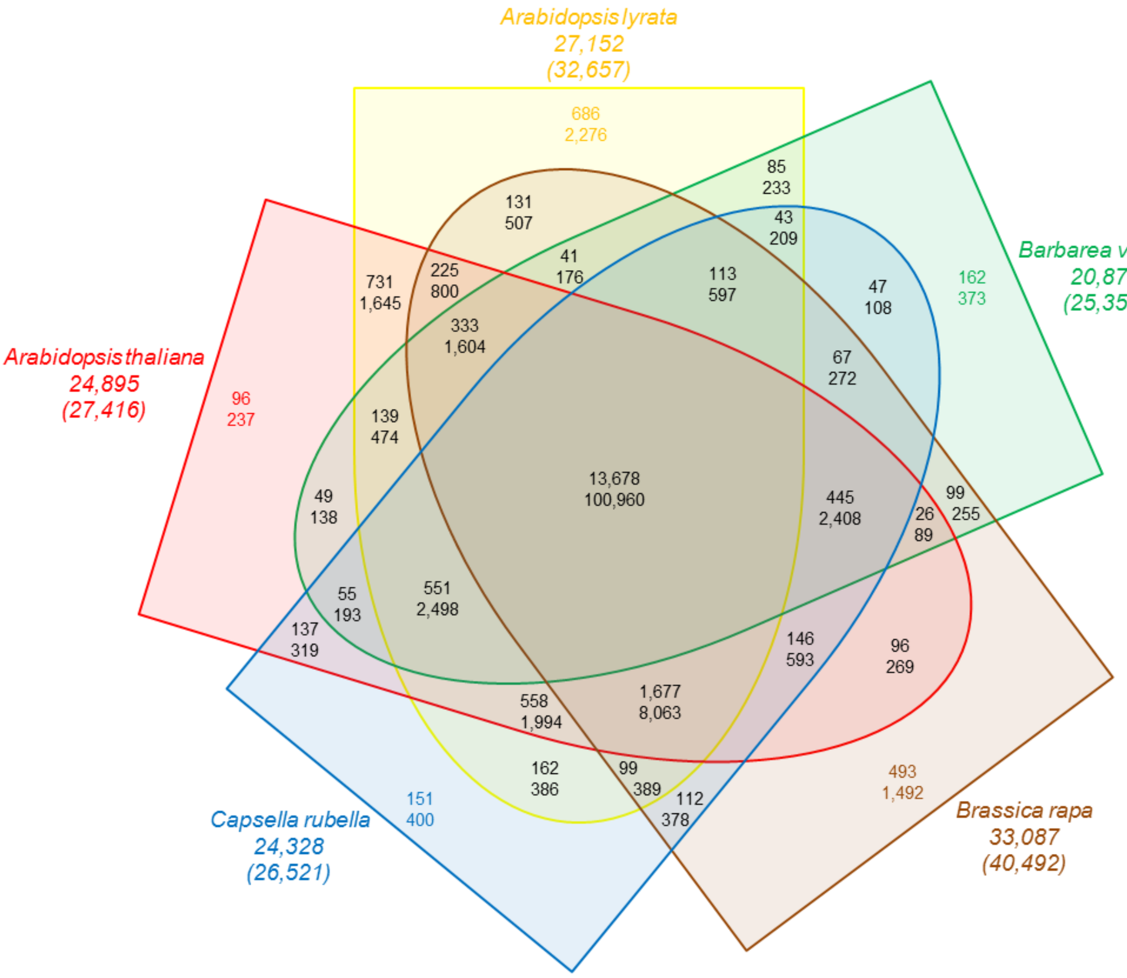

Supplementary Figure 5: Venn diagram of orthologous groups of genes. Proteins from five species (*A. thaliana, A. lyrata, B.vulgaris, C.rubella, and B.rapa*) were used to generate the Venn diagram after OrthoMCL analysis. Top number shows the number of orthologous groups, and the bottom the total number of genes in these orthologous groups.

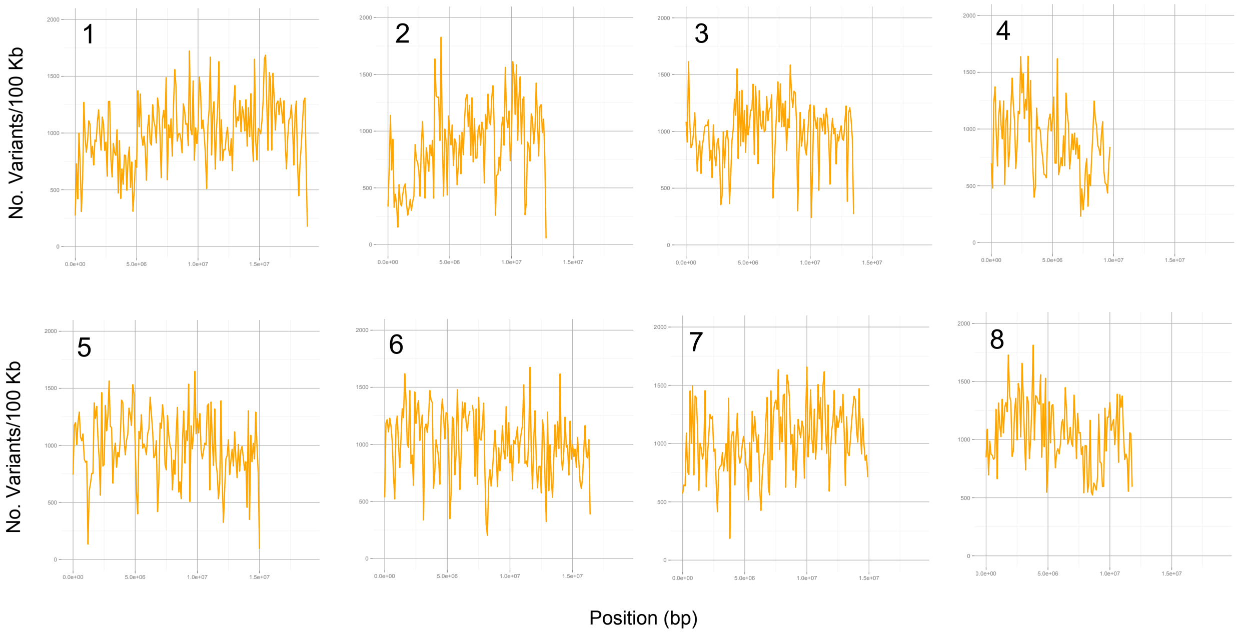

Supplementary Figure 6: Distribution of fixed differences between *B. vulgaris* P and G-type. The frequency of fixed differences in bins of 100 Kb along the eight linkage groups of *B. vulgaris.*

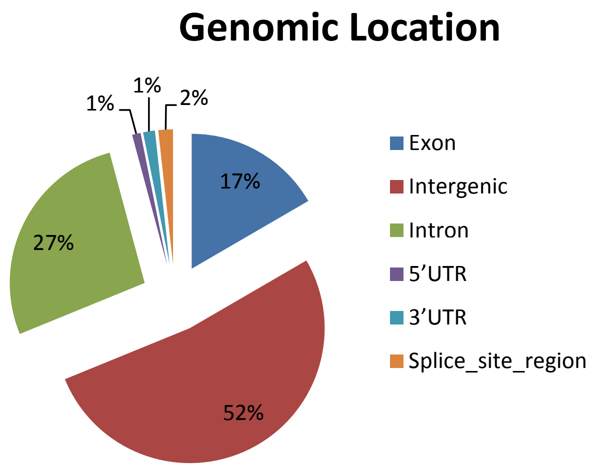

Supplementary Figure 7: Genome location of fixed differences between B. vulgaris P and G-type. The distribution of fixed differences according to genomic feature.

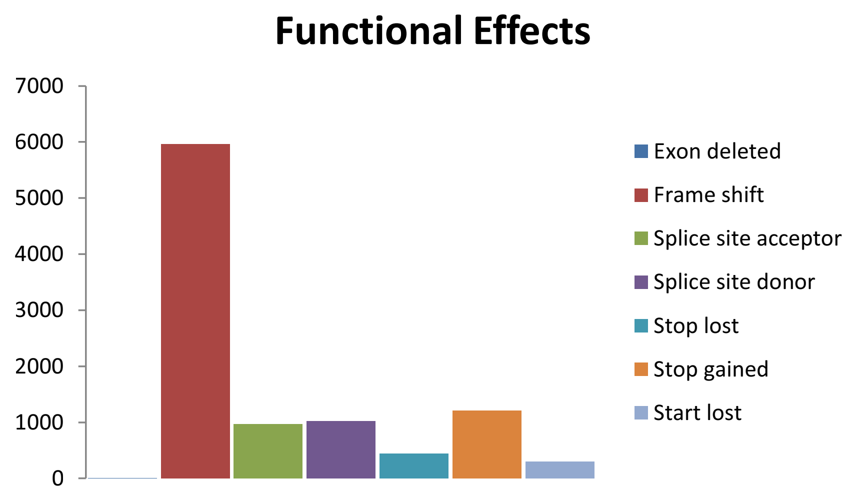

Supplementary Figure 8:Functional effects of fixed differences between B. vulgaris P and G-types. The distribution of fixed differences according to genomic feature.

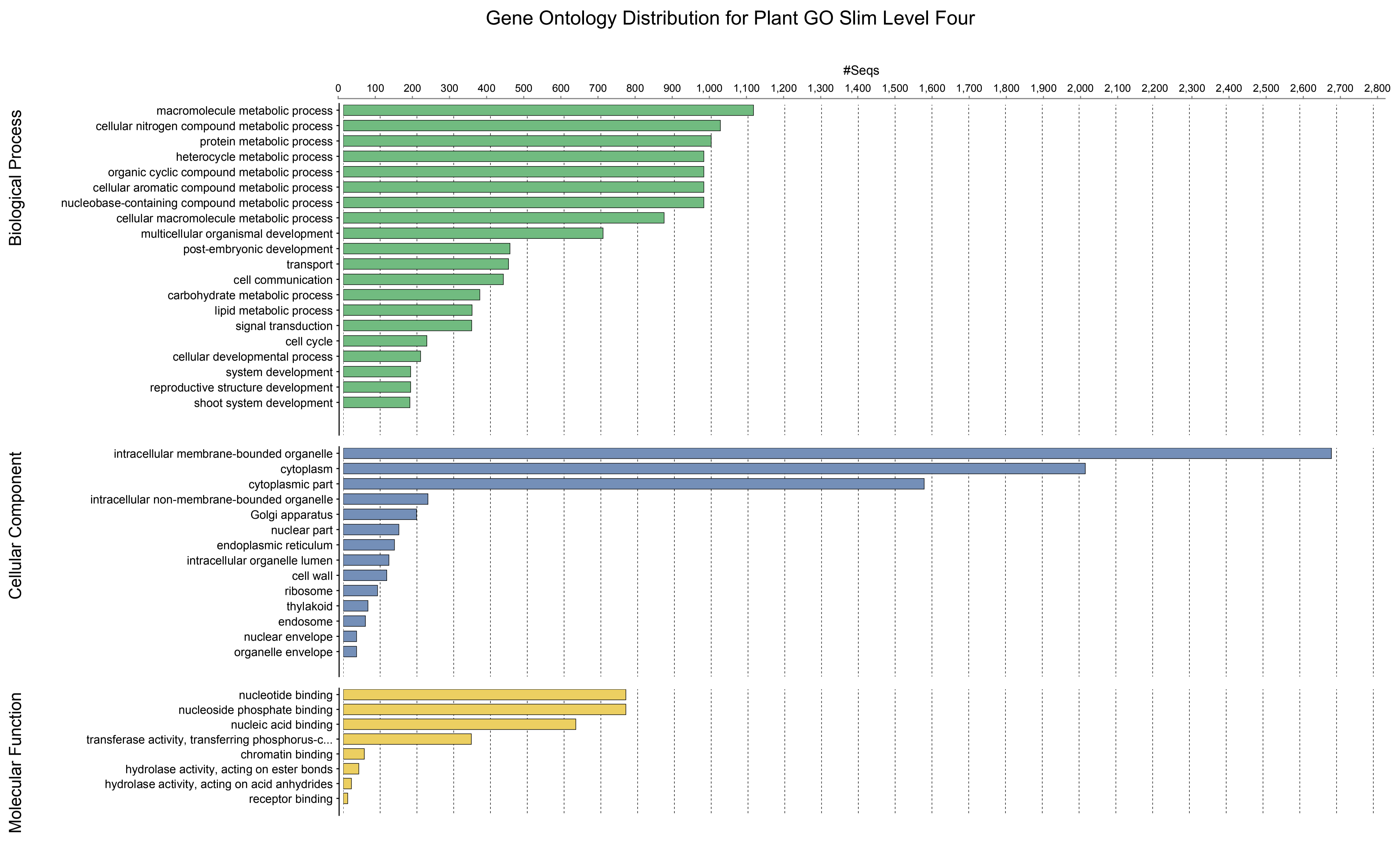

Supplementary Figure 9: Gene ontology distribution for genes with high impact fixed differences. Gene ontology distribution for a plant GO slim file on level four, organized into biological process, cellular component, and mlecular function.

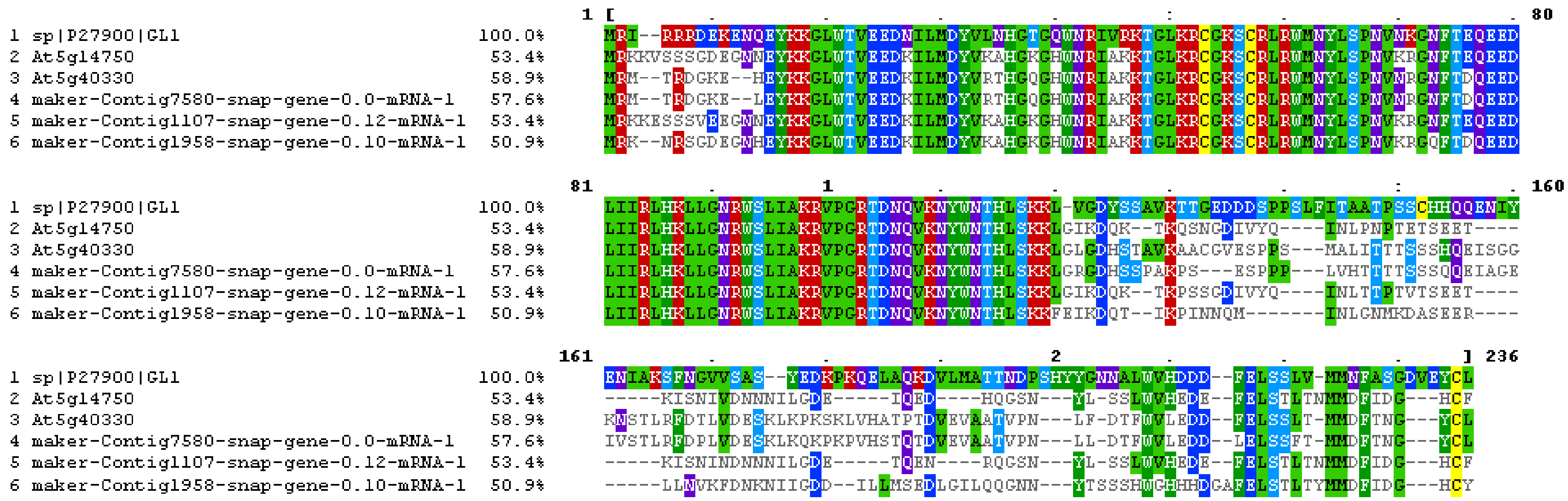

Supplementary Figure 10: Alignments of GL1 sequence homologs. Visulaisation of gapped alignments generated by Muscle for genes in *A. thaliana* and *B.vulgaris* that were homologus to GL1. These sequences correpsond to those used in the phylogentic analysis shown in Figure 1(D).

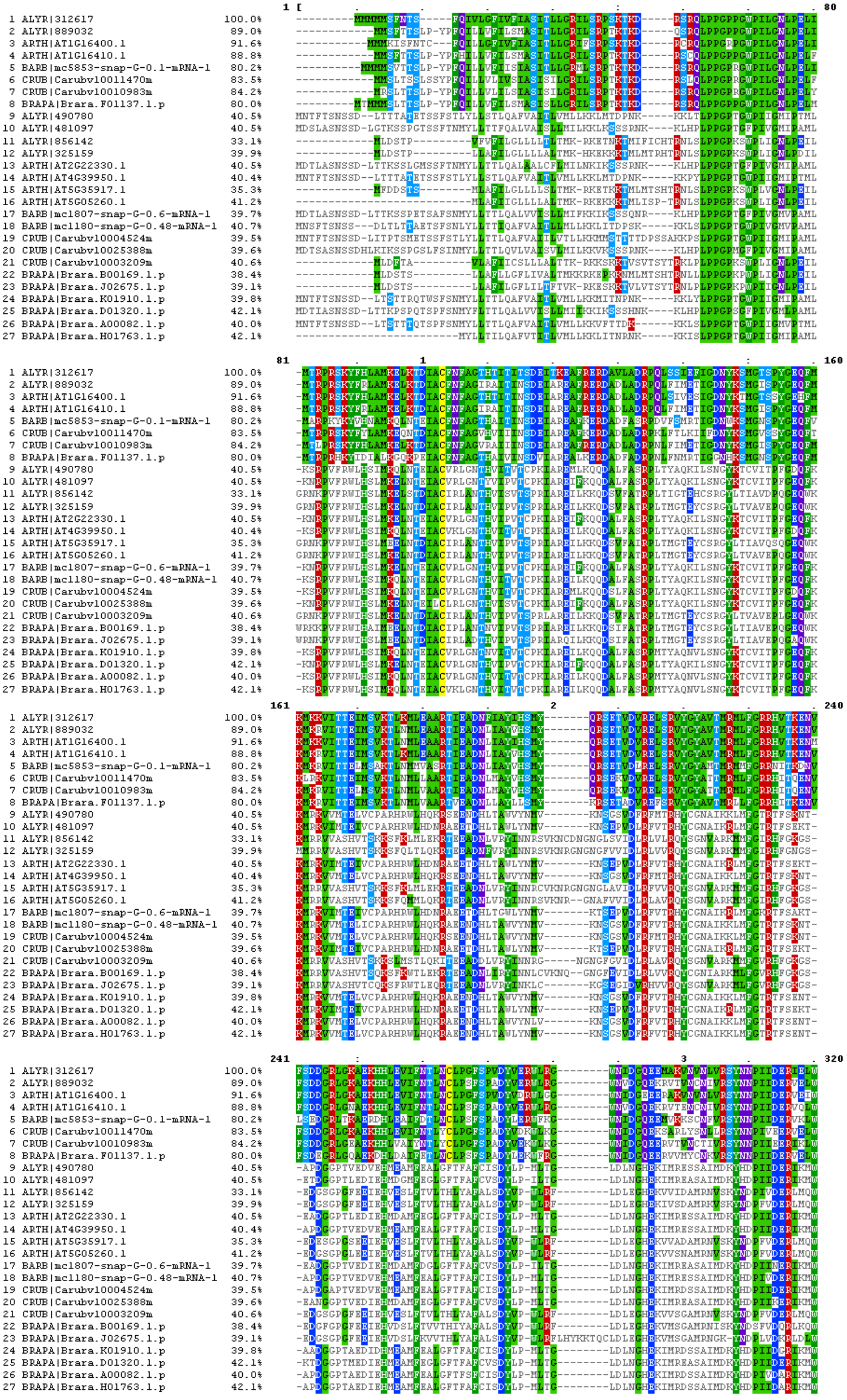

Supplementary Figure 11: Alignments of CYP79 sequence homologs. Visulisation of gapped alignments generated by Muscle for genes homologous to CYP79F2 from *A. thaliana*. These sequences correspond to those used in the phylogentic analysis shown in Figure 2.

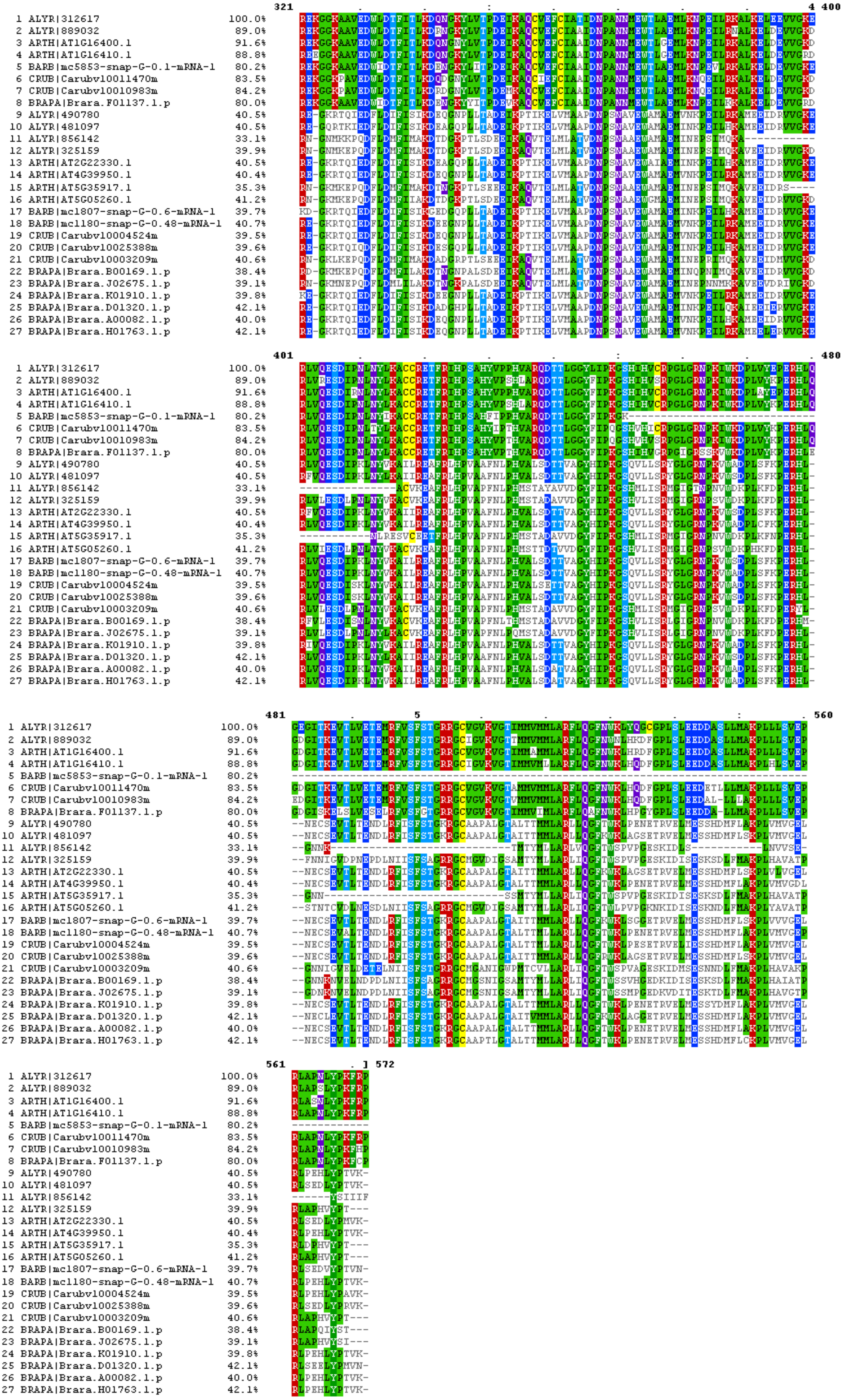

Supplementary Figure 11 continued: Alignments of CYP79 sequence homologs. Visulisation of gapped alignments generated by Muscle for genes homologous to CYP79F2 from *A. thaliana*. These sequences correspond to those used in the phylogentic analysis shown in Figure 2.

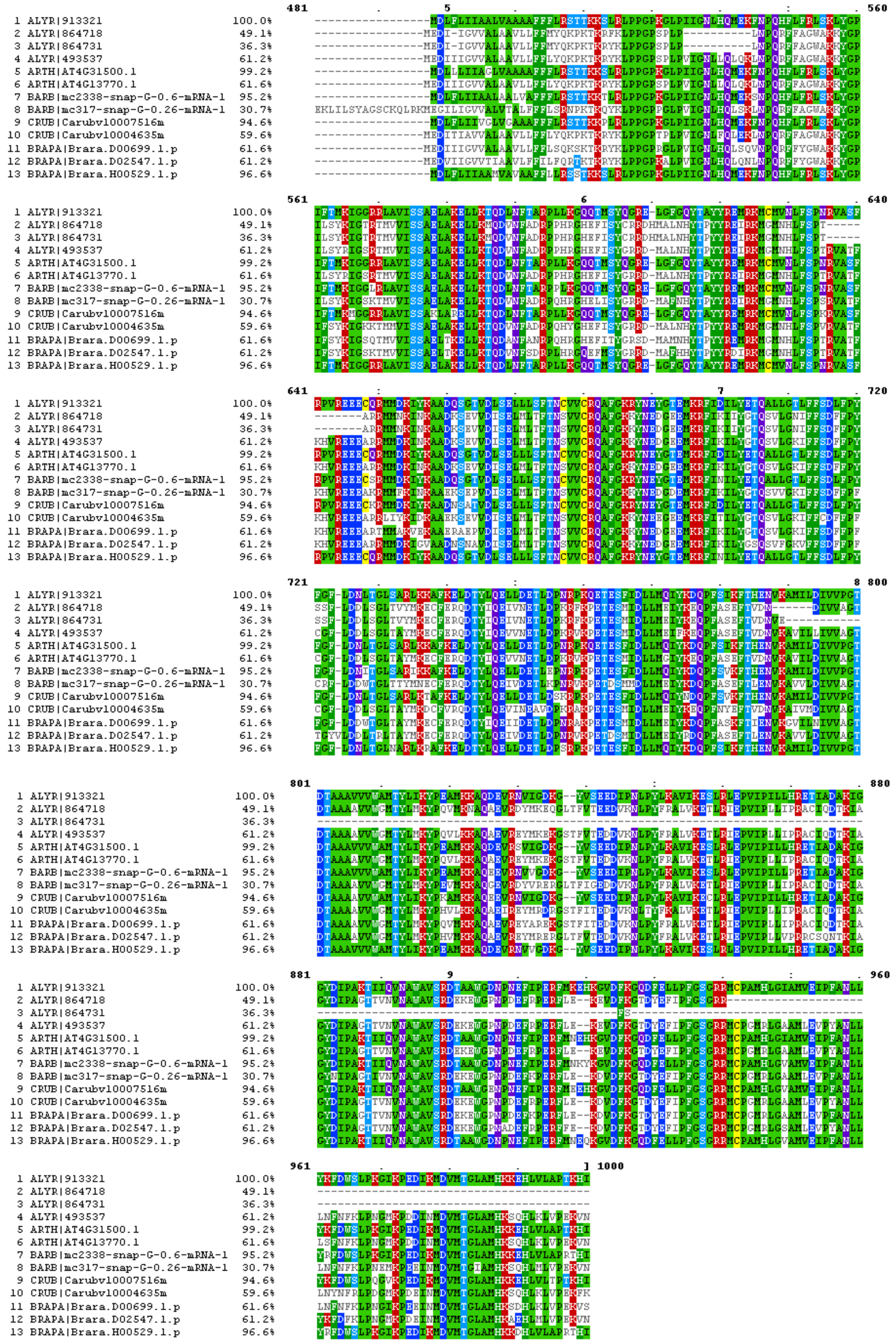

Supplementary Figure 12: Alignments of CYP83 sequence homologs. Visulisation of gapped alignments generated by Muscle for genes homologous to CYP83A1 from *A. thaliana*. The start of sequence 8 was truncated. These sequences correspond to those used in the phylogentic analysis shown in Figure 2.

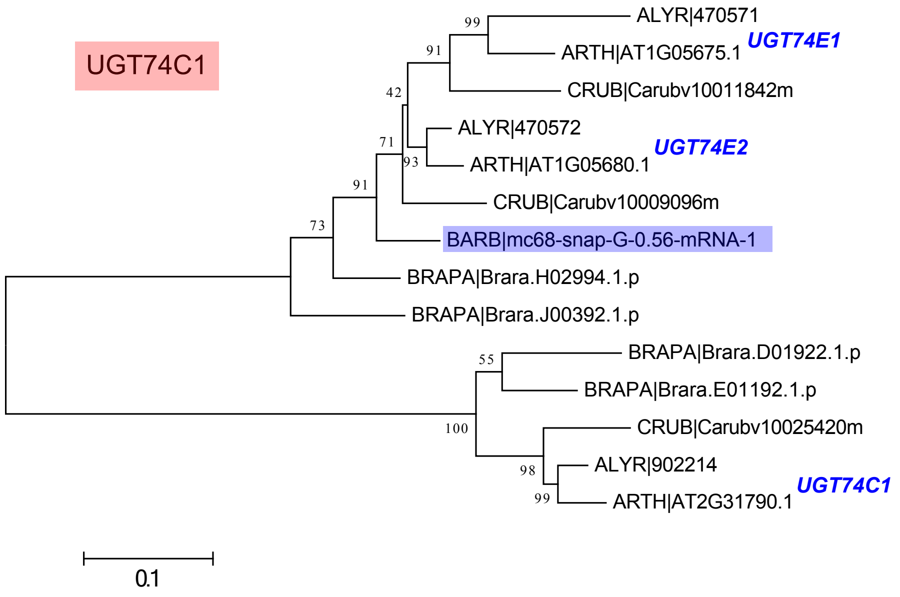

Supplementary Figure 13: Phylogenetic analysis of UGT74C1 homologs. Molecular phylogenetic analysis by the Maximum-Likelihood method using JTT matrix-based model. Bootstrap values are shown next to brances. The tree is mid-point rooted, drawn to scale, with branch lengths proportional to the number of substitutions per site. We analysed genes from the orthologous group containing the A. thaliana UGT74C1 gene. *B. vulgaris* proteins are highlighted in blue.

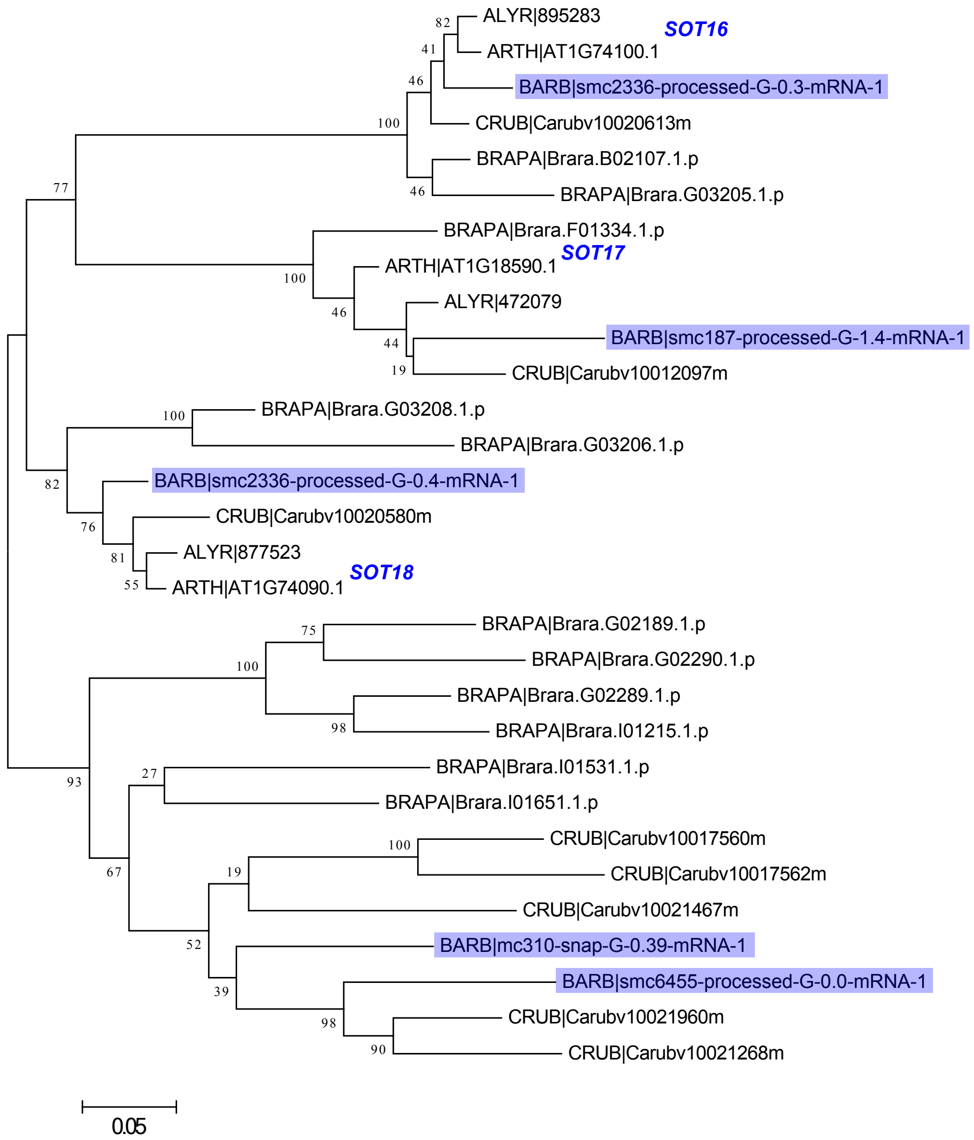

Supplementary Figure 14: Phylogenetic analysis of SOT homologs. Molecular phylogenetic analysis by the Maximum-Likelihood method using JTT matrix-based model. Bootstrap values are shown next to brances. The tree is mid-point rooted, drawn to scale, with branch lengths proportional to the number of substitutions per site. We analysed genes from the orthologous group containing the *A. thaliana* SOT gene. *B. vulgaris* proteins are highlighted in blue.

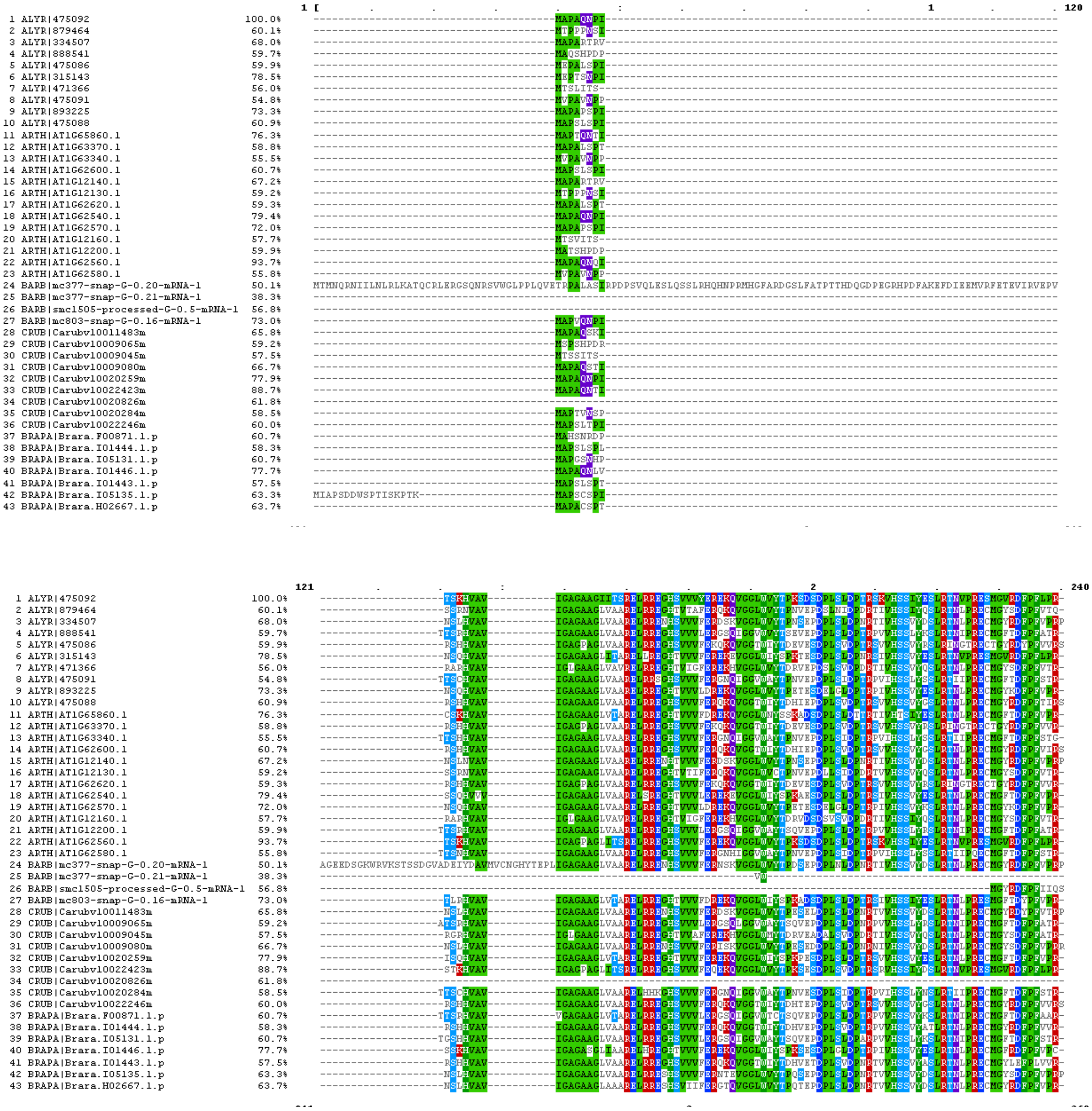

Supplementary Figure 15: Alignments of FMOS-GSOX sequence homologs. Visulisation of gapped alignments generated by Muscle for genes homologous to FMO-GSOX1 from *A. thaliana*. These sequences correspond to those used in the phylogentic analysis shown in Figure 3.

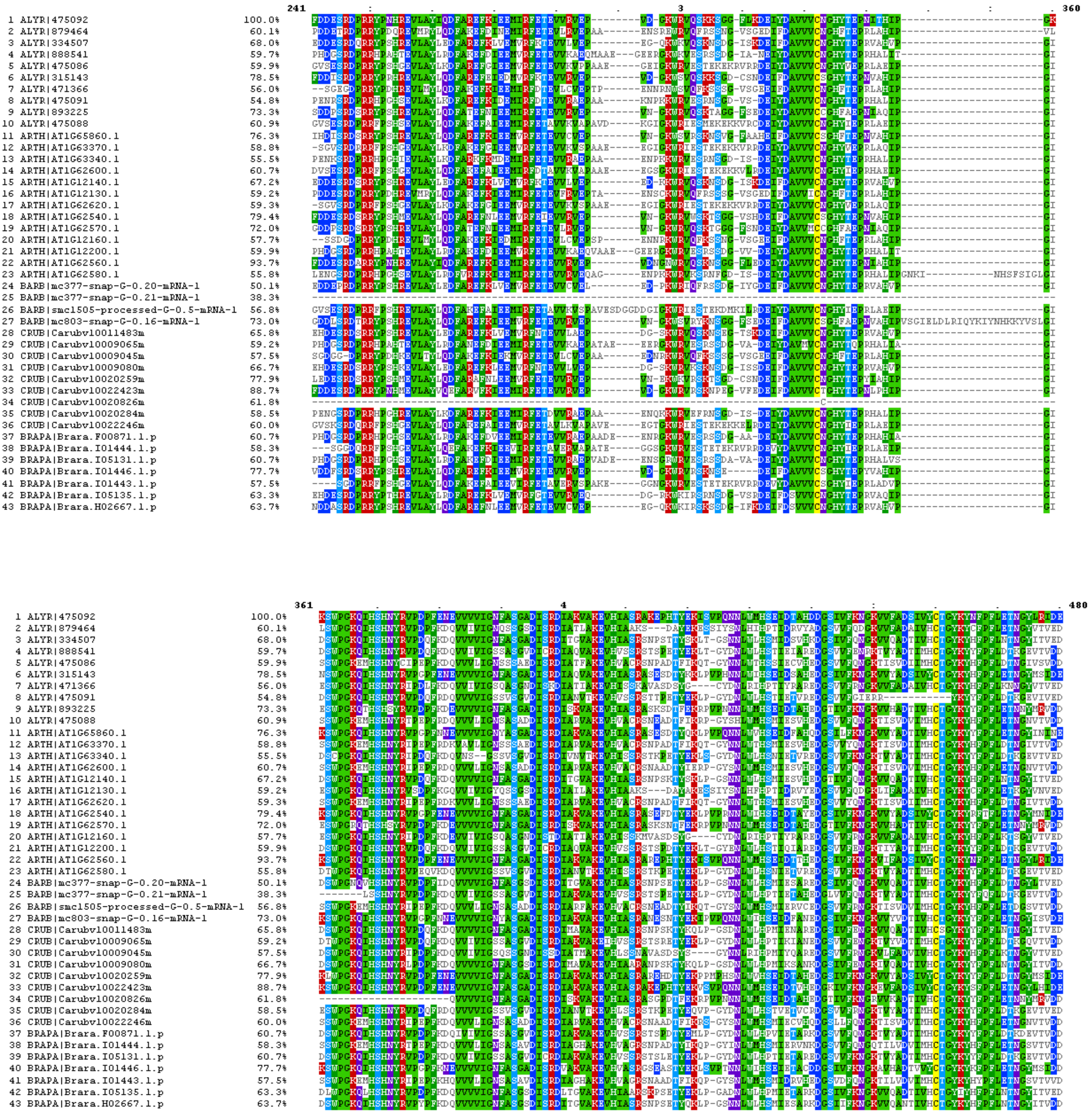

Supplementary Figure 15 continued: Alignments of FMOS-GSOX sequence homologs. Visulisation of gapped alignments generated by Muscle for genes homologous to FMO-GSOX1 from *A. thaliana*. These sequences correspond to those used in the phylogentic analysis shown in Figure 3.

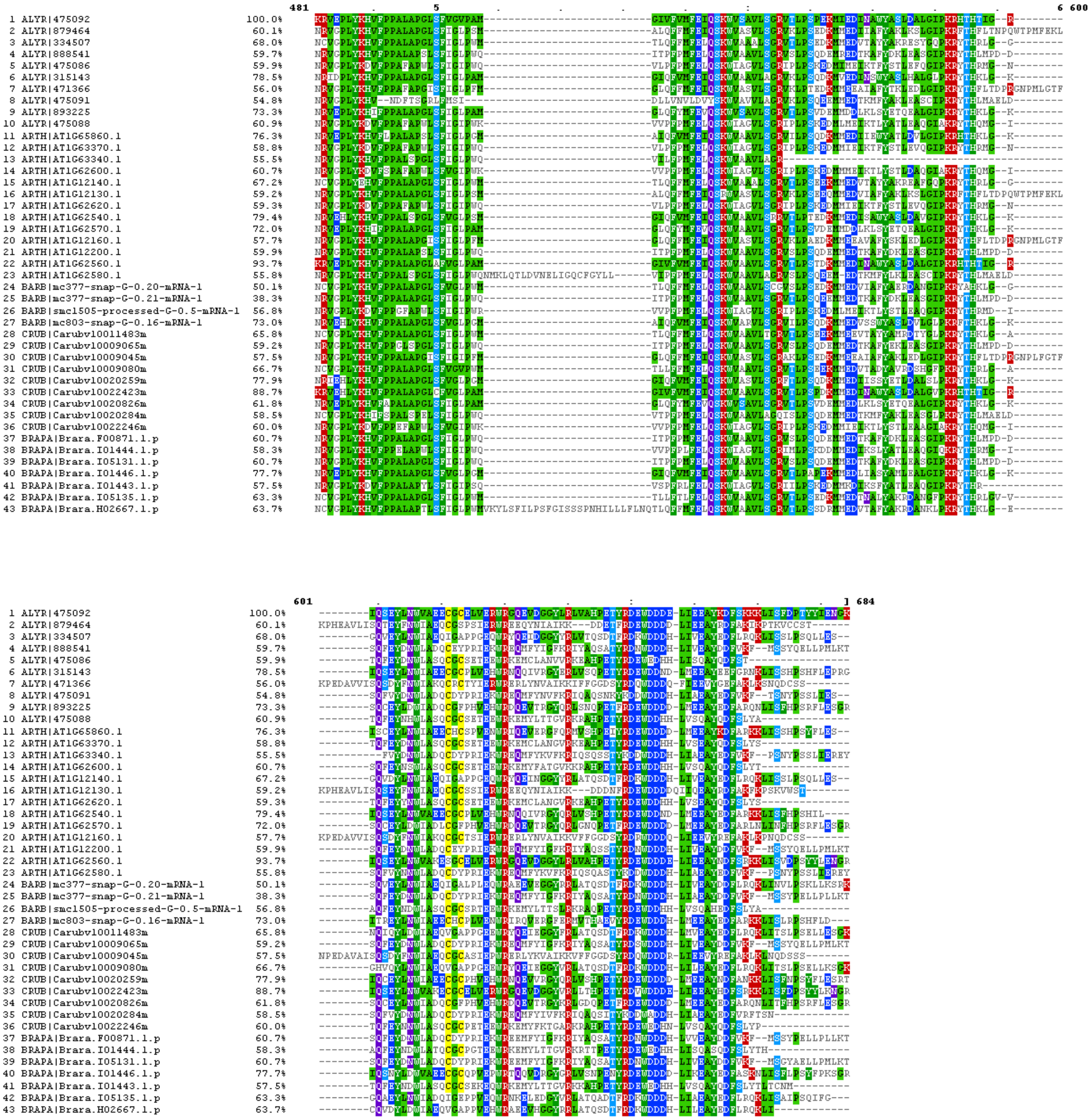

Supplementary Figure 15 continued: Alignments of FMOS-GSOX sequence homologs. Visulisation of gapped alignments generated by Muscle for genes homologous to FMO-GSOX1 from *A. thaliana*. These sequences correspond to those used in the phylogentic analysis shown in Figure 3.

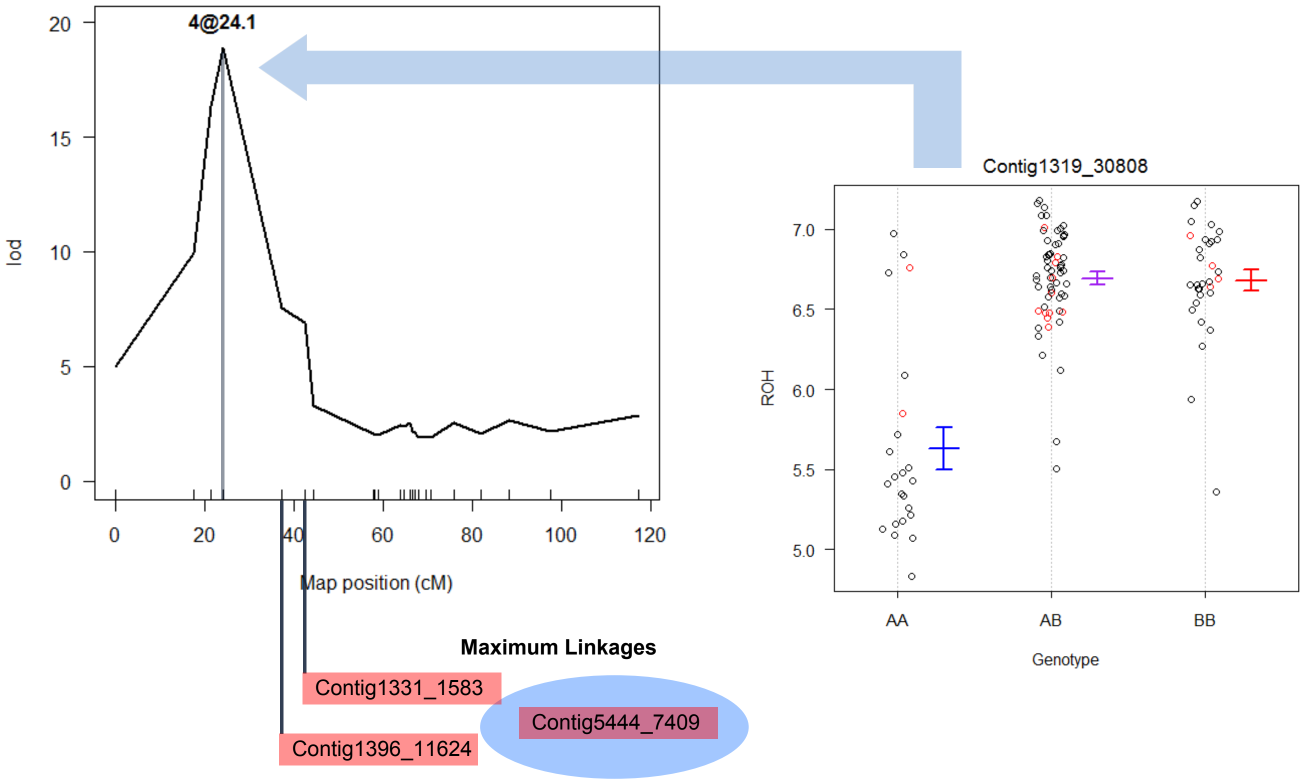

Supplementary Figure 16: QTL for epiglucobarbarin production. Location of QTL for epiglucobarbarin on linkage group four. We highlight the two SNPs that had the strongest linkage to an SNP in the scaffold (Contig5444) containing the BvGS-OH-like 2 gene (LOD scores of 14.69 and 11.64).

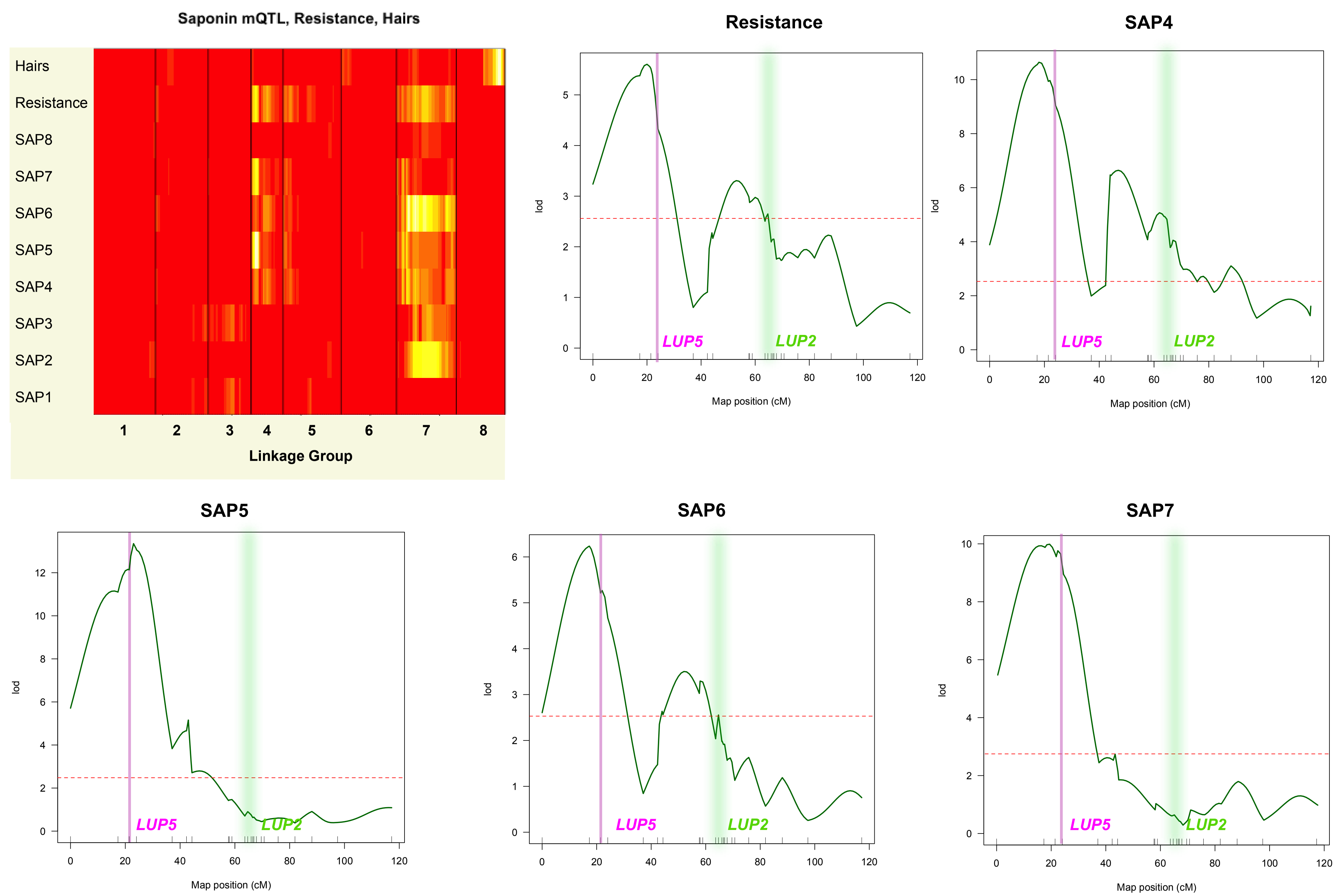

Supplementary Figure 17: QTL for various traits mapped in an F2 *B. vulgaris* population. LOD heatmap showing mapping of the following traits; 8 saponins, insect resistance and hairs (top left). Remaining graphs are LOD plots for a selection of traits with QTL on linkage group four. The minimum LOD threshold after 1000 permutations is shown on each plot. The position of LUP5 and the most likely approximate position of LUP2 are also shown on each plot.

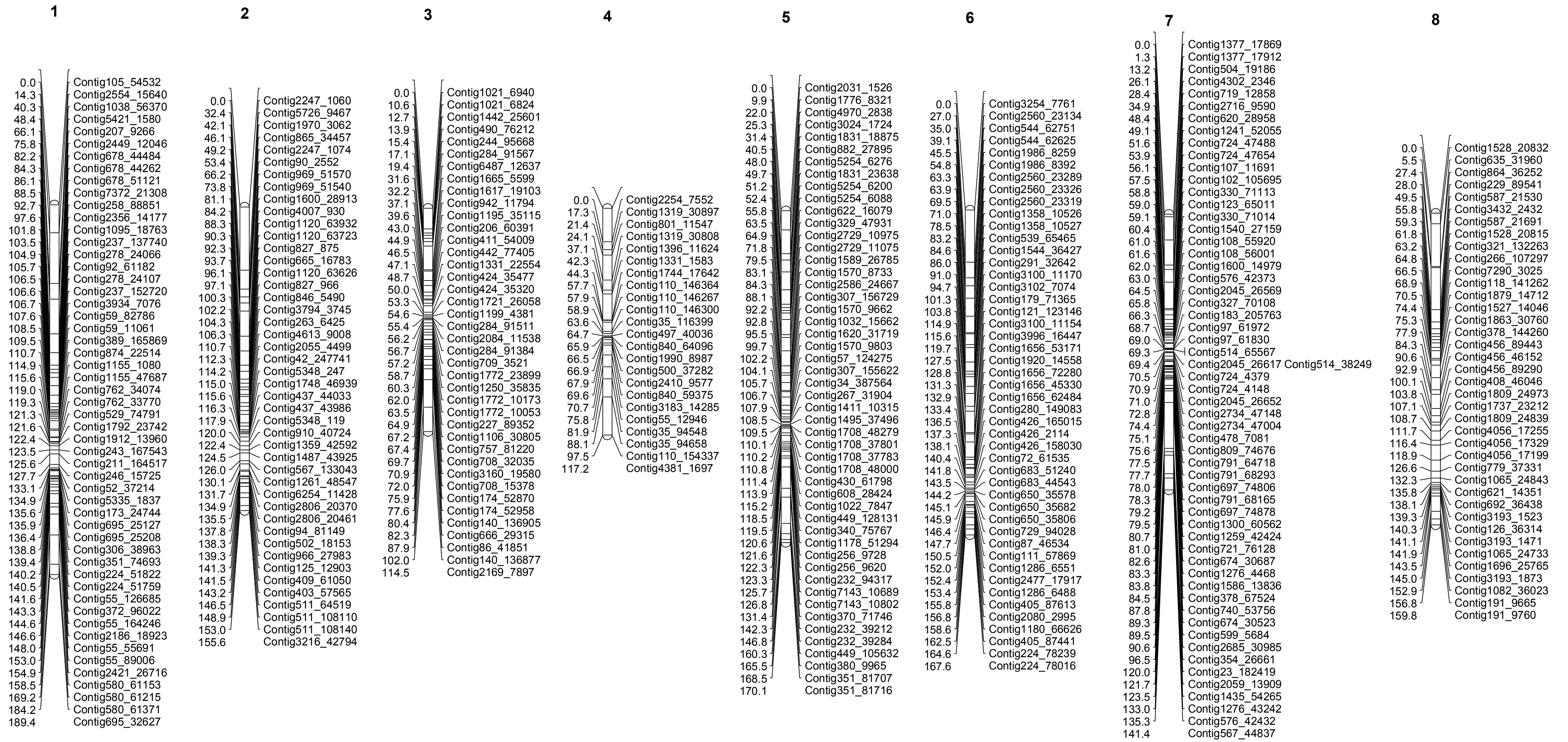

Supplementary Figure 18: Slim downed genetic linkage map of F2 population. Genetic linkage map generated from SNPs that were identified using genotyping-by-sequencing of an F2 population. This is a slimmed down version of the map in supplemental figure S3, and was used for QTL analysis. It remvoes some of the redundancy present in the previous map.

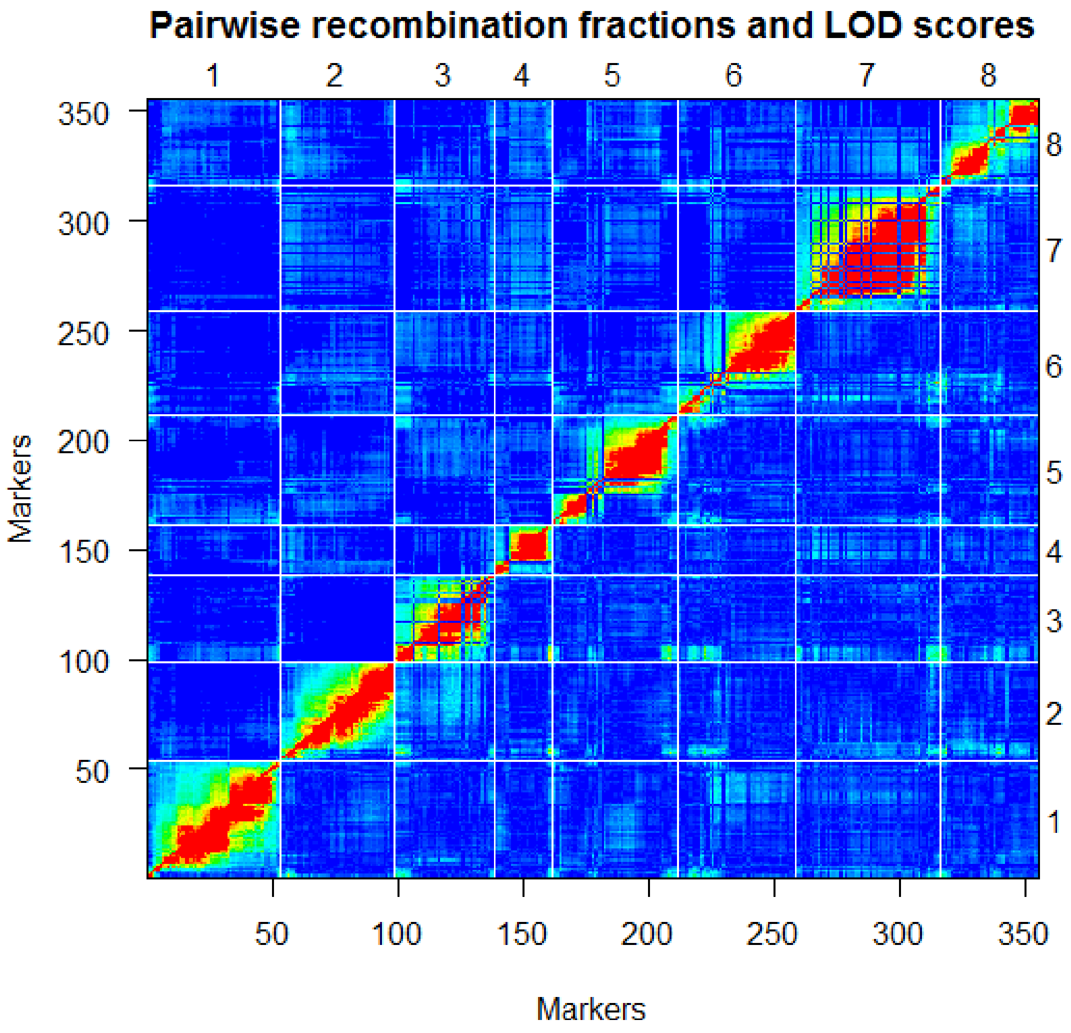

Supplementary Figure 19: Pairwise recombination fractions and LOD scores. Recombination fractions are shown in the upper left triangle, and LOD scores are shown in the lower right triangle. Red correpsonds to a large LOD or a small recombination fraction, while blue is the reverse. We observe little evidence of linkage between markers across linkage groups.

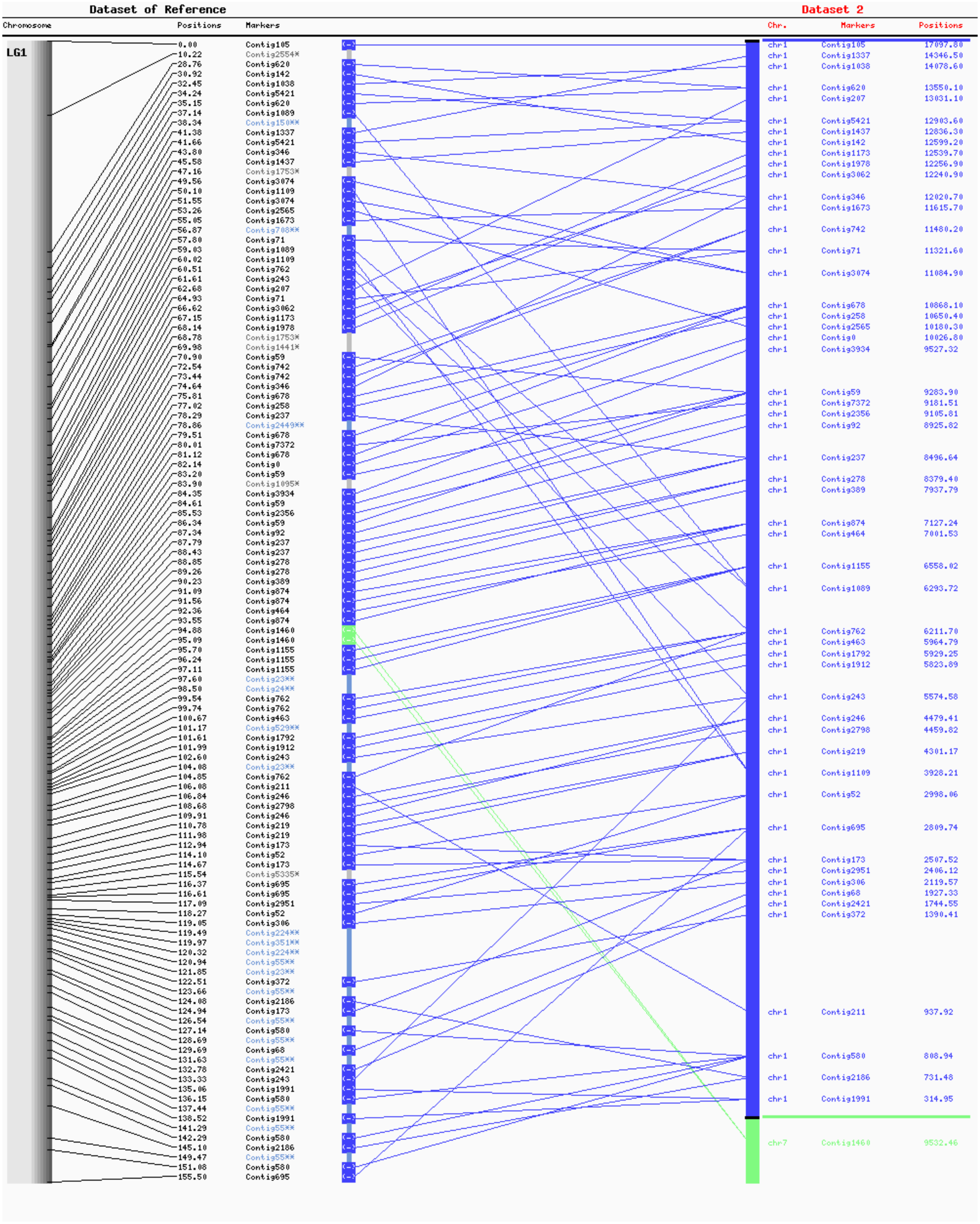

Supplementary Figure 20: Order of markers on LG1 and predicted position within the *A. lyrata* genome. The markers on the *B. vulgaris* genome (left) are located within annotated contigs. One-to-one puative orthologs between both species were identified, which allowed the *B. vulgaris* markers to be located within the A. lyrata genome (right). Position within the *A. lyrata* genome are shown in Mb.

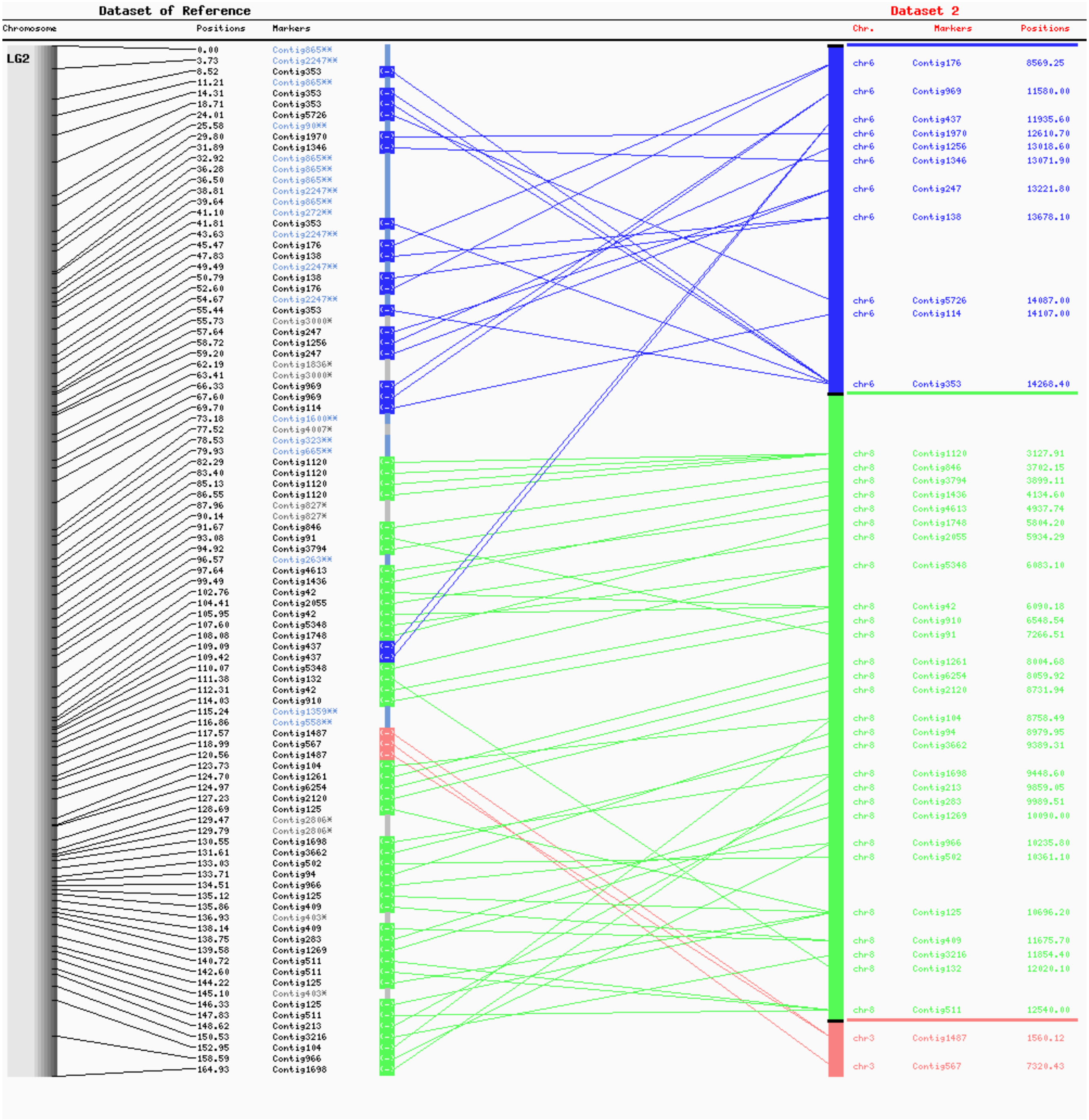

Supplementary Figure 21: Order of markers on LG2 and predicted position within the *A. lyrata* genome. The markers on the *B. vulgaris* genome (left) are located within annotated contigs. One-to-one puative orthologs between both species were identified, which allowed the *B. vulgaris* markers to be located within the A. lyrata genome (right). Position within the *A. lyrata* genome are shown in Mb.

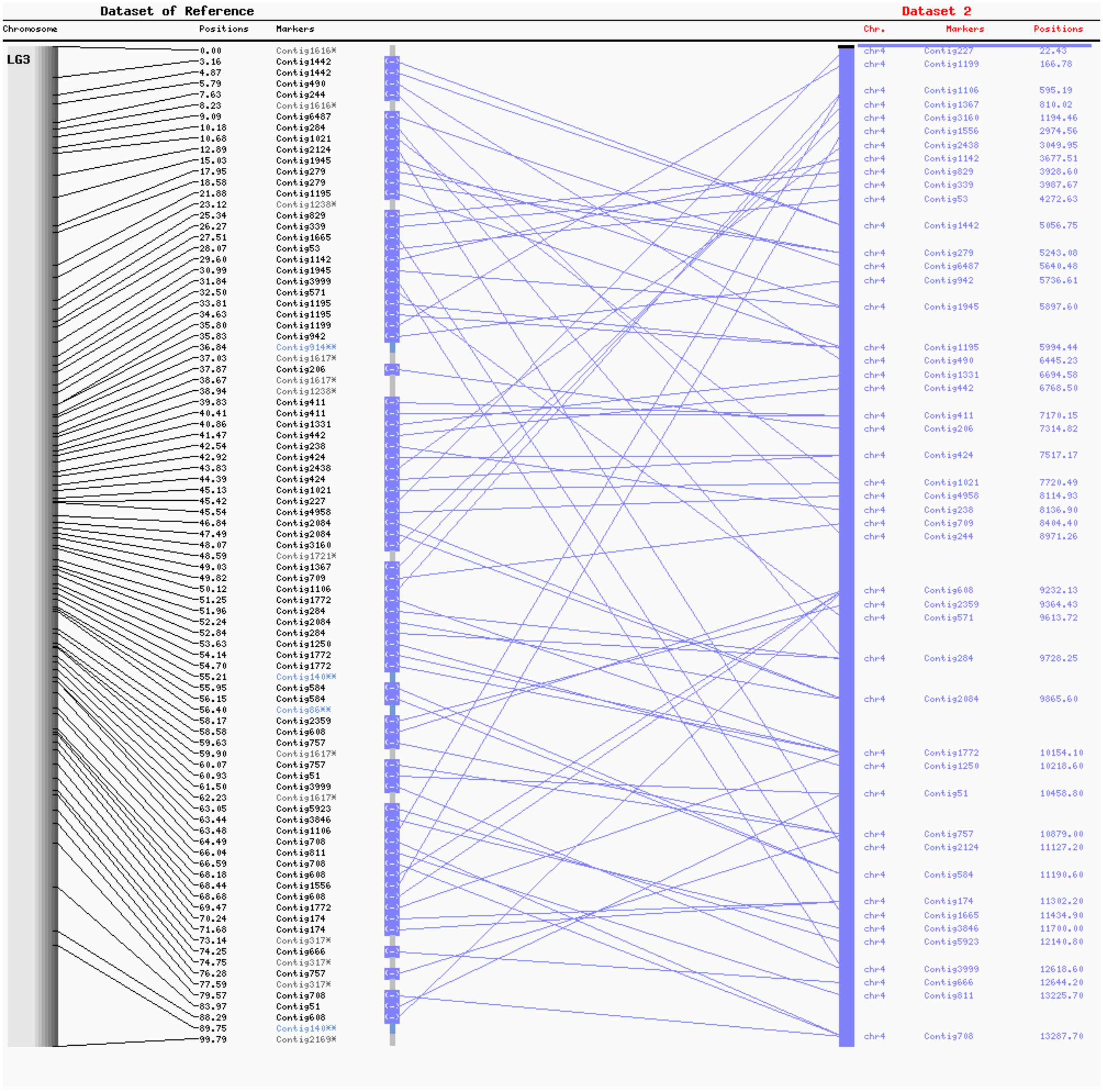

Supplementary Figure 22: Order of markers on LG3 and predicted position within the *A. lyrata* genome. The markers on the *B. vulgaris* genome (left) are located within annotated contigs. One-to-one puative orthologs between both species were identified, which allowed the *B. vulgaris* markers to be located within the A. lyrata genome (right). Position within the *A. lyrata* genome are shown in Mb.

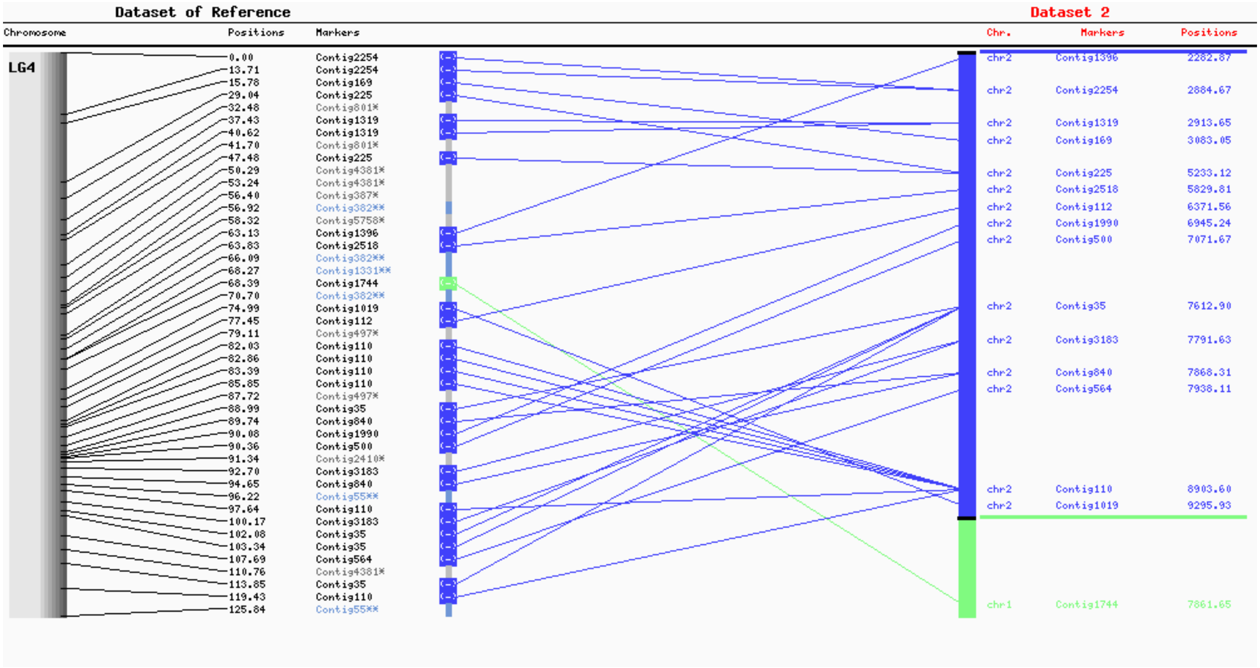

Supplementary Figure 23: Order of markers on LG4 and predicted position within the *A. lyrata* genome. The markers on the *B. vulgaris* genome (left) are located within annotated contigs. One-to-one puative orthologs between both species were identified, which allowed the *B. vulgaris* markers to be located within the A. lyrata genome (right). Position within the *A. lyrata* genome are shown in Mb.

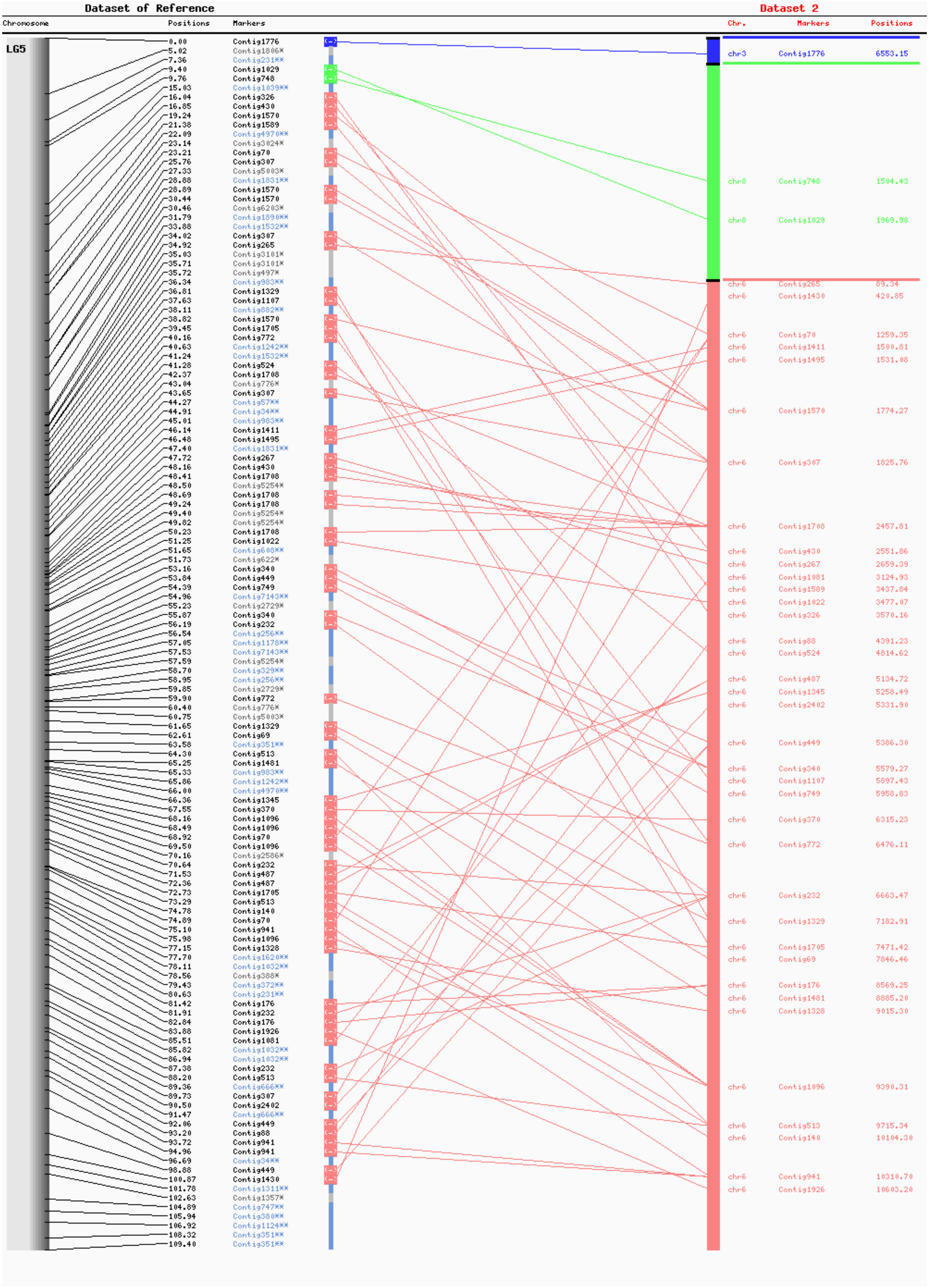

Supplementary Figure 24: Order of markers on LG5 and predicted position within the *A. lyrata* genome. The markers on the *B. vulgaris* genome (left) are located within annotated contigs. One-to-one puative orthologs between both species were identified, which allowed the *B. vulgaris* markers to be located within the A. lyrata genome (right). Position within the *A. lyrata* genome are shown in Mb.

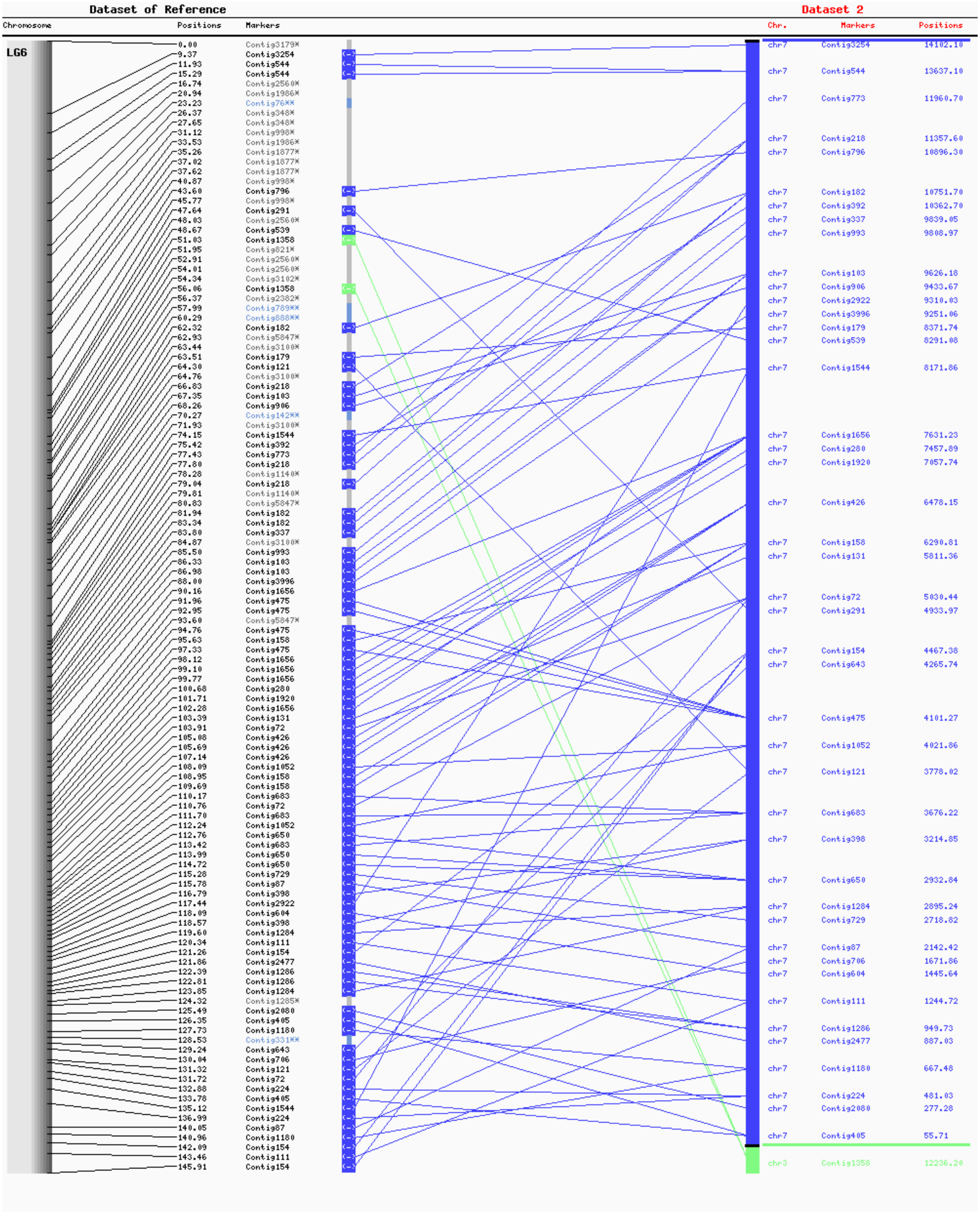

Supplementary Figure 25: Order of markers on LG6 and predicted position within the *A. lyrata* genome. The markers on the *B. vulgaris* genome (left) are located within annotated contigs. One-to-one puative orthologs between both species were identified, which allowed the *B. vulgaris* markers to be located within the A. lyrata genome (right). Position within the *A. lyrata* genome are shown in Mb.

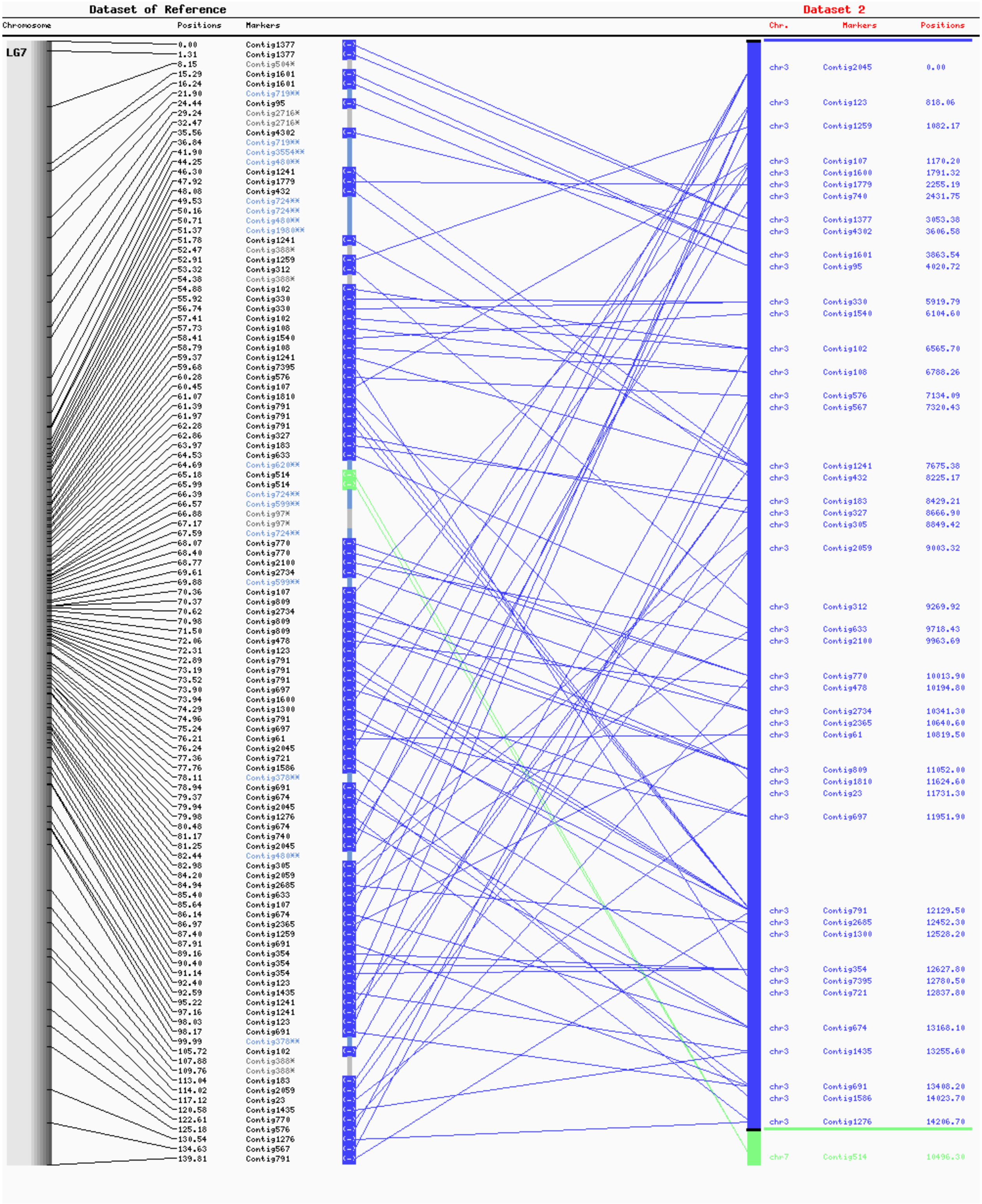

Supplementary Figure 26: Order of markers on LG7 and predicted position within the *A. lyrata* genome. The markers on the *B. vulgaris* genome (left) are located within annotated contigs. One-to-one puative orthologs between both species were identified, which allowed the *B. vulgaris* markers to be located within the A. lyrata genome (right). Position within the *A. lyrata* genome are shown in Mb.

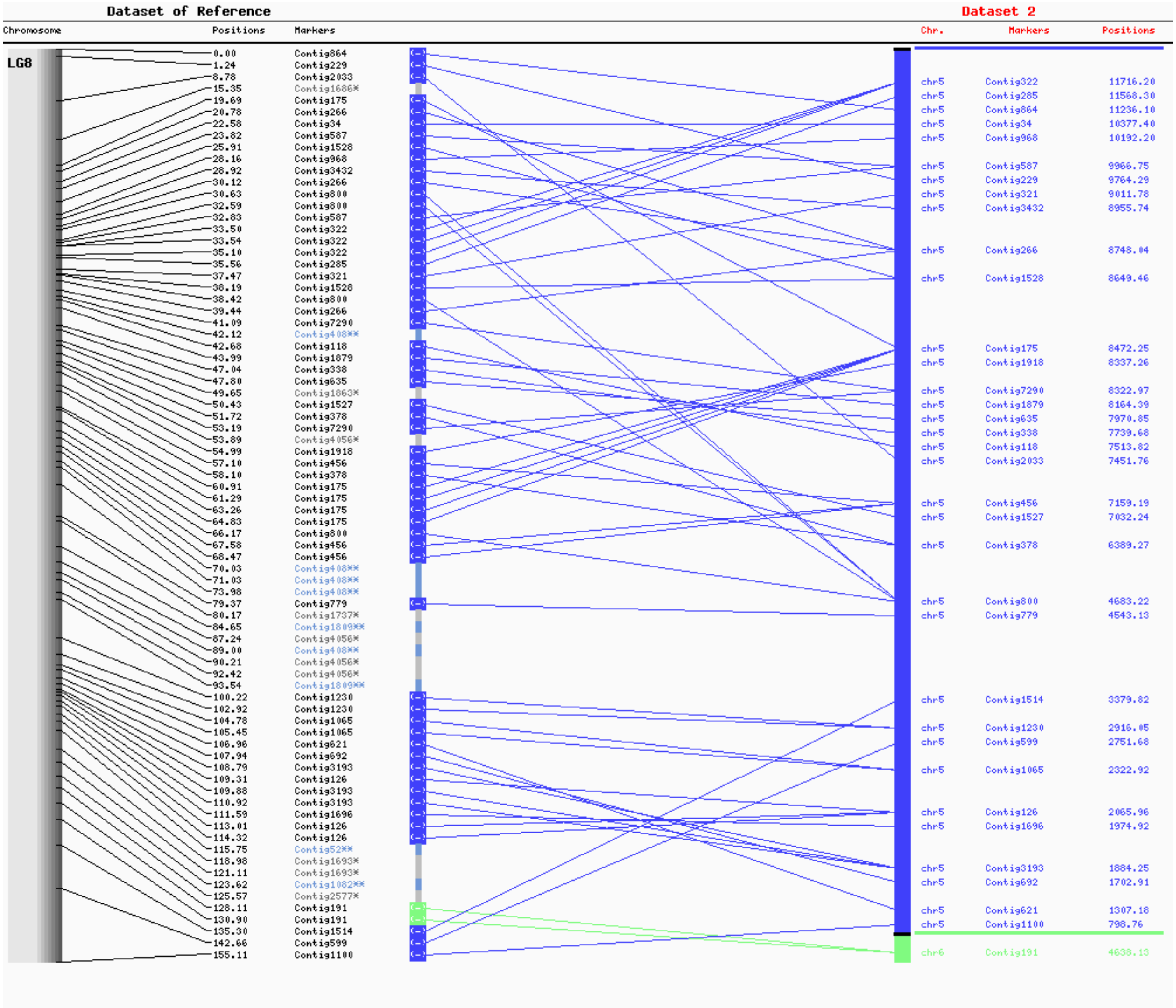

Supplementary Figure 27: Order of markers on LG8 and predicted position within the *A. lyrata* genome. The markers on the *B. vulgaris* genome (left) are located within annotated contigs. One-to-one puative orthologs between both species were identified, which allowed the *B. vulgaris* markers to be located within the A. lyrata genome (right). Position within the *A. lyrata* genome are shown in Mb.

A. lyrata Chr1

bp

bp

Contig678

Supplementary Figure 28: Comparison of Contig678 with a region from A. lyrata chromosome 1. Genes within B. vulgaris Contig678 were used in a BLAST against A. lyrata chromosome 1 and only matches with and evalue < 0.0001 and a minimum query coverage of 40% are shown.
The position of these genes along B. vulgaris chromsome 1 is shown on top.

A. lyrata Chr1

bp

bp

Contig59

Supplementary Figure 29: Comparison of Contig59 with a region from A. lyrata chromosome 1. Genes within B. vulgaris Contig59 were used in a BLAST against A. lyrata chromosome 1 and only matches with and evalue < 0.0001 and a minimum query coverage of 40% are shown.
The position of these genes along B. vulgaris chromsome 1 is shown on top.

A. lyrata Chr1

bp

bp

Contig278

Supplementary Figure 30: Comparison of Contig278 with a region from A. lyrata chromosome 1. Genes within B. vulgaris Contig278 were used in a BLAST against A. lyrata chromosome 1 and only matches with and evalue < 0.0001 and a minimum query coverage of 40% are shown.
The position of these genes along B. vulgaris chromsome 1 is shown on top.

Supplementary Tables

Supplemental Table S1: Overview of data types used in the generation of a *B. vulgaris* G-type draft genome assembly.

Technology	Library Type	Reads	Yield (Gbp)
**Illumina**	‘200’ bp PE	50.8 million pairs	9.5
**Illumina**	‘500’ bp PE	38.9 million pairs	7.2
**Illumina**	LJD ‘20’ Kb	66.0 million pairs	11.6
**PacBio**	10 Kbp: 1 x 120 min movie	3.8 million	5.25

Supplemental Table S2: Statistics based on BLAT alignments of de-novo assembled G-type transcriptome to the reference assembly.

Statistic	Ratio
Ratio of transcripts with BLAT entry	0.97 (39787/41018)
Total % coverage of all positions	0.96 (35618947/36907888)
No. transcripts mapping to a single scaffold	0.88 (34933/39787)
Average no. scaffolds per mapped transcript	1.18

Supplemental Table S3: Statistics from CEGMA analysis evaluating genome completeness.

	No. Proteins	% Completeness	Total	Average	%Ortho
**Complete**	**237**	**95.56**	**455**	**1.92**	**53.16**
Group 1	65	98.48	105	1.62	40.00
Group 2	51	91.07	99	1.94	54.90
Group 3	58	95.08	111	1.91	58.62
Group 4	63	96.92	140	2.22	60.32
**Partial**	**244**	**98.39**	**526**	**2.16**	**59.84**
Group 1	66	100.00	112	1.70	43.94
Group 2	55	98.21	123	2.24	63.64
Group 3	60	98.36	131	2.18	61.67
Group 4	63	96.92	160	2.54	71.43

Supplemental Table S4: Summary of genetic linkage map incorporating all available marker data.

	LG1	LG2	LG3	LG4	LG5	LG6	LG7	LG8
No. Markers	117	94	90	44	130	115	115	77
Length (cM)	155.5	164.9	99.8	125.8	109.4	145.9	139.8	155.1

Supplemental Table S5: Summary of final two QTL model for hairiness.

```
Method: Haley-Knott regression
Model:  normal phenotype
Number of observations : 113

Full model result
----------------------------------
Model formula: y ~ Q1 + Q2

       df        SS        MS      LOD     %var Pvalue(Chi2)    Pvalue(F)
Model   4  99589.59 24897.398 10.23488 34.10513 1.430415e-09 3.207475e-09
Error 108 192418.05  1781.649
Total 112 292007.64


Drop one QTL at a time ANOVA table:
----------------------------------
        df Type III SS   LOD   %var F value Pvalue(Chi2) Pvalue(F)
4@57.7   2       22701 2.736  7.774   6.371        0.002   0.00242 **
8@143.5  2       87104 9.163 29.829  24.445        0.000  1.75e-09 ***
---
Signif. codes:  0 '***' 0.001 '**' 0.01 '*' 0.05 '.' 0.1 ' ' 1
```

Supplemental Table S6: Summary of single QTL model for glucobarbarin.

```
Method: Haley-Knott regression
Model:  normal phenotype
Number of observations : 115

Full model result
----------------------------------
Model formula: y ~ Q1

       df       SS          MS      LOD     %var Pvalue(Chi2)    Pvalue(F)
Model   2 29.37472 14.6873577 12.47754 39.32647 3.329559e-13 7.046586e-13
Error 112 45.31980  0.4046411
Total 114 74.69451
```

Supplemental Table S7: Summary of single QTL model for epiglucobarbarin.

```
Method: Haley-Knott regression
Model:  normal phenotype
Number of observations : 115

Full model result
----------------------------------
Model formula: y ~ Q1

       df       SS          MS     LOD     %var Pvalue(Chi2) Pvalue(F)
Model   2 20.64906 10.3245287 18.8946 53.07565            0         0
Error 112 18.25590  0.1629991
Total 114 38.90495
```
